# Low Diversity and Instability of the Sinus Microbiota over Time in Adults with Cystic Fibrosis

**DOI:** 10.1128/spectrum.01251-22

**Published:** 2022-09-12

**Authors:** Catherine R. Armbruster, Kelvin Li, Megan R. Kiedrowski, Anna C. Zemke, Jeffrey A. Melvin, John Moore, Samar Atteih, Adam C. Fitch, Matthew DuPont, Christopher D. Manko, Madison L. Weaver, Jordon R. Gaston, John F. Alcorn, Alison Morris, Barbara A. Methé, Stella E. Lee, Jennifer M. Bomberger

**Affiliations:** a Department of Microbiology and Molecular Genetics, University of Pittsburgh grid.412689.0grid.21925.3d School of Medicine, Pittsburgh, Pennsylvania, USA; b Center for Medicine and the Microbiome, University of Pittsburgh grid.412689.0grid.21925.3d and University of Pittsburgh Medical Center, Pittsburgh, Pennsylvania, USA; c Department of Medicine, University of Alabama at Birmingham, Birmingham, Alabama, USA; d Department of Medicine, Division of Pulmonary, Allergy, and Critical Care Medicine, University of Pittsburgh grid.412689.0grid.21925.3d School of Medicine, Pittsburgh, Pennsylvania, USA; e Department of Otolaryngology, University of Pittsburgh Medical Center, Pittsburgh, Pennsylvania, USA; f Department of Pediatrics, Children’s Hospital of Pittsburgh, Pittsburgh, Pennsylvania, USA; University of Manitoba

**Keywords:** *Pseudomonas aeruginosa*, *Staphylococcus*, chronic rhinosinusitis, cystic fibrosis, inflammation, microbiota, polymicrobial, respiratory pathogens, sinus

## Abstract

Chronic rhinosinusitis (CRS) is a common, yet underreported and understudied manifestation of upper respiratory disease in people with cystic fibrosis (CF). Recently developed standard of care guidelines for the management of CF CRS suggest treatment of upper airway disease may ameliorate lower airway disease. We sought to determine whether changes to sinus microbial community diversity and specific taxa known to cause CF lung disease are associated with increased respiratory disease and inflammation. We performed 16S rRNA gene sequencing, supplemented with cytokine analyses, microscopy, and bacterial culturing, on samples from the sinuses of 27 adults with CF CRS. At each study visit, participants underwent endoscopic paranasal sinus sampling and clinical evaluation. We identified key drivers of microbial community composition and evaluated relationships between diversity and taxa with disease outcomes and inflammation. Sinus community diversity was low, and the composition was unstable, with many participants exhibiting alternating dominance between Pseudomonas aeruginosa and staphylococci over time. Despite a tendency for dominance by these two taxa, communities were highly individualized and shifted composition during exacerbation of sinus disease symptoms. Exacerbations were also associated with communities dominated by Staphylococcus spp. Reduced microbial community diversity was linked to worse sinus disease and the inflammatory status of the sinuses (including increased interleukin-1β [IL-1β]). Increased IL-1β was also linked to worse sinus endoscopic appearance, and other cytokines were linked to microbial community dynamics. Our work revealed previously unknown instability of sinus microbial communities and a link between inflammation, lack of microbial community diversity, and worse sinus disease.

**IMPORTANCE** Together with prior sinus microbiota studies of adults with CF chronic rhinosinusitis, our study underscores similarities between sinus and lower respiratory tract microbial community structures in CF. We show how community structure tracks with inflammation and several disease measures. This work strongly suggests that clinical management of CRS could be leveraged to improve overall respiratory health in CF. Our work implicates elevated IL-1β in reduced microbiota diversity and worse sinus disease in CF CRS, suggesting applications for existing therapies targeting IL-1β. Finally, the widespread use of highly effective cystic fibrosis transmembrane conductance regulator (CFTR) modulator therapy has led to less frequent availability of spontaneous expectorated sputum for microbiological surveillance of lung infections. A better understanding of CF sinus microbiology could provide a much-needed alternative site for monitoring respiratory infection status by important CF pathogens.

## INTRODUCTION

The upper airways are constantly exposed to microbes inhaled from the environment. In healthy individuals, these microbes are captured by mucus produced by the sinonasal epithelium and removed by mucocilliary clearance, but this process is impaired in people with the genetic disorder cystic fibrosis (CF) (1). The sinonasal cavity is thought to be the first site in the respiratory tract to be colonized by opportunistically pathogenic microbes that may seed downstream lung disease in CF (2). Chronic rhinosinusitis (CRS) is defined as symptomatic chronic infection and inflammation of the sinonasal cavity. CRS is common among people with CF, yet underreported, and the interactions between microbes in the upper respiratory tract, local inflammatory responses, and clinical outcomes are poorly understood.

The unified airway hypothesis is a conceptual framework originating in the field of asthma research that links upper (URT) and lower (LRT) respiratory tract disease (3). This framework proposes that treatment of URT symptoms can improve LRT disease and vice versa. A growing body of literature supports similarities and interplay between CRS and LRT disease in CF. For example, the microbiota of CF CRS resembles that of the LRT in terms of the taxa present and diversity (4–6); children harbor comparatively diverse microbes, whereas adults tend to be dominated by one or very few organisms (7). Furthermore, medical or surgical management of sinus disease symptoms may lead to better LRT outcomes in CF (8, 9). Recently, studies by our team and others have shown that evolved traits and evolutionary strategies of Pseudomonas aeruginosa isolated from the sinuses of people with CF CRS resemble those previously reported among CF lung populations (10, 11). We have also shown that sinus exacerbation increases the odds of a subsequent pulmonary exacerbation (12). Likewise, others have shown that sinonasal quality of life worsens during CF LRT exacerbations (13). Together, these studies strongly suggest that CF CRS impacts LRT disease. Recently developed standard of care guidelines for the management of CF CRS also support this notion (14). One gap in the CF CRS literature is our lack of understanding regarding whether and how CF sinus communities change over time as the conditions in the surrounding host environment change (e.g., during periods of increased inflammation and/or exacerbation of symptoms).

The goal of this study was to evaluate how the microbial composition and inflammatory environment of the sinuses relate to upper and lower airway disease in adults with CF CRS. We hypothesized that lack of sinus microbial community diversity and changes in relative abundance of opportunistic pathogens or pathobionts (commensals that can cause disease under certain circumstances) would be associated with increased sinus disease severity and inflammation. Our study reveals similarities between microbiota-related correlates of CF sinus disease and inflammation to those described for the lower respiratory tract in CF. It also supports the necessity for further investigation into therapies targeting microbe-immune interactions and highlights the relevance of sinus disease to overall CF respiratory health.

## RESULTS

### Cohort demographics and association of CF-related diabetes (CFRD) with lower respiratory disease.

We performed a longitudinal study of 33 adults with CF and symptomatic CRS who had undergone prior functional endoscopic sinus surgery (FESS) as a treatment for CF CRS (Table 1) (12). During quarterly clinic visits and unscheduled visits due to exacerbation of clinical symptoms, we obtained at least one endoscopically guided specimen for 16S amplicon sequencing from 27 of the 33 study participants. Additional samples included sinus secretions that were collected for inflammatory cytokine analyses, bacterial culturing, and microscopy. An average of five longitudinal microbiota samples were collected during study visits from 18 of 27 participants (range, 2 to 10 study visits with samples), whereas cross-sectional samples were collected from 9 of 27 participants. The median length of follow-up for the study participants with longitudinal samples was 522 days (range, 91 to 770 days). The following demographic and clinical characteristics of the cohort (covariates) were controlled for in most of the later analyses: patient identification (ID) (to control for repeated measures within study participants), age, sex, CFTR mutation class, diagnosis of CF-related diabetes (CFRD), body mass index (BMI), and topical antibiotic use. We tested for associations between these covariates and patient outcomes examined throughout this study, including sinus exacerbation, pulmonary exacerbation, sinus disease score (Sino-nasal Outcome Test [SNOT-22] [15] or modified Lund-Kennedy [mLK] [16]), and lung function (median forced expiratory volume in 1 s [FEV_1_]), independent of information on the microbiota. We found that CFRD was associated with reduced FEV_1_ (regression coefficient, −68.3; Holm-Bonferroni adjusted *P* value of <0.05). No other covariates were significantly associated with the outcomes of interest.

**TABLE 1:** Demographics of the adult CF CRS microbiota study cohort^*a*^

Parameter	Result for microbiota cohort (*n* = 27/33)
Longitudinal microbiota samples, no. (%)	18/27 (66.6)
Median age on enrollment, yr (range)	27.6 (19.7–43.6)
Male, no. (%)	9/27 (33.3)
CFTR genotype, no. (%)	
ΔF508 homozygous	13/27 (48.2)
ΔF508/other	11/27 (40.7)
Other/other	2/27 (7.4)
Missing	1/27 (3.7)
CFRD (%)	12/27 (44.4)
No. (%) using:	
Topical sinus antibiotic	22/27 (81.5)
Topical or oral steroid	21/27 (77.7)
CFTR corrector/modulator	10/27 (37)

a The cohort includes 27 people from the larger 33-person study, for whom we sequenced at least one 16S amplicon microbiota sample from a paranasal sinus swab collected by endoscope. For 18 of these 27 people, we sequenced at least two samples, giving us longitudinal information. Drug use is reported for any time during study. CF-related diabetes (CFRD) is reported for at any time within the study or ±12 months of enrollment. Clinical parameters of the full cohort (*n* = 33) were published by Zemke et al. (12).

### Low diversity and instability of sinus microbial communities in adults with CF CRS.

**FIG 1:**
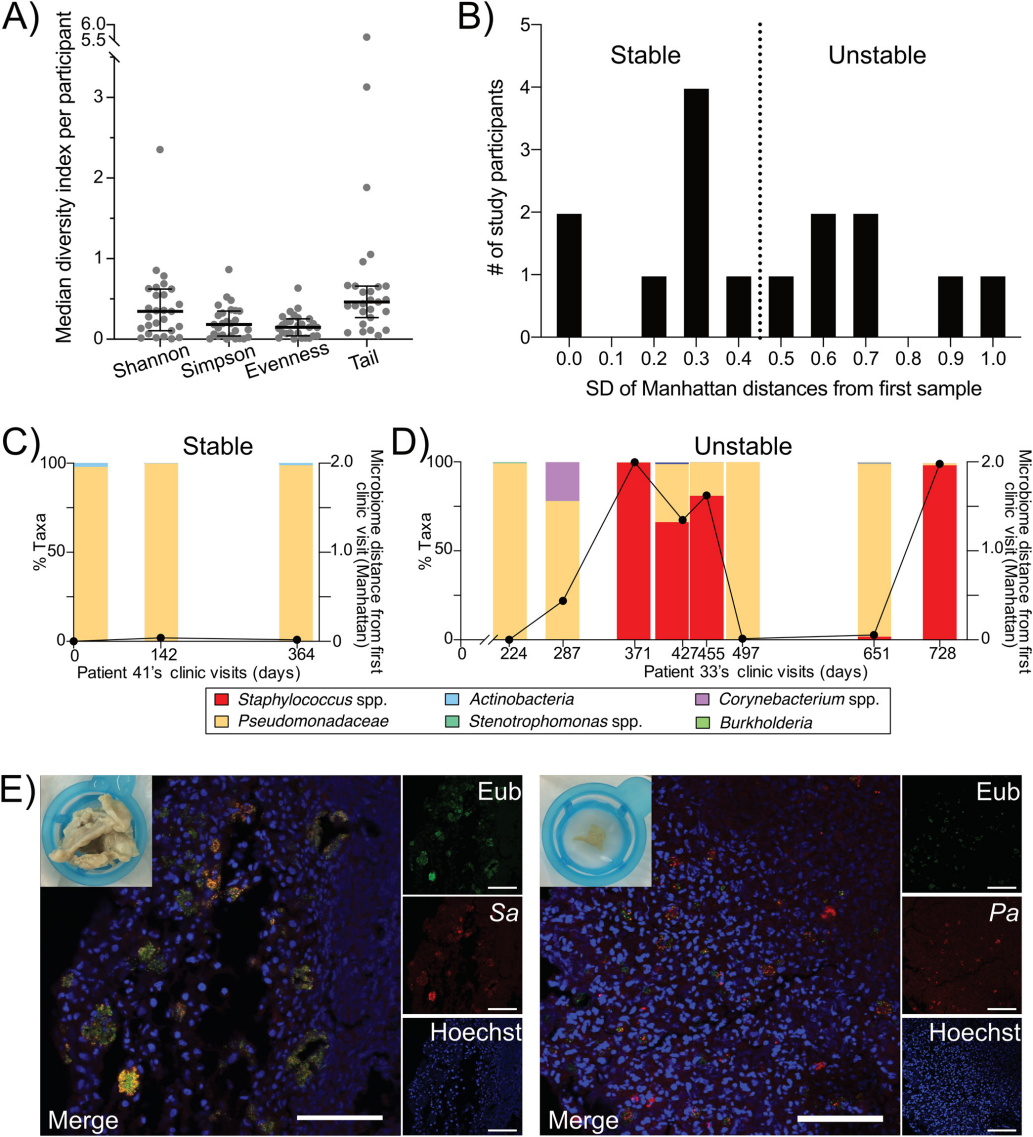
Sinus microbial community diversity is low, and the composition can be unstable in adults with CF chronic rhinosinusitis, with most study participants’ sinuses dominated by P. aeruginosa or Staphylococcus spp. (A) Box plots depicting the median Shannon or Simpson diversity indices, evenness, or Tail statistic per study participant. Overlaid are the cohort’s median and interquartile range. *n* = 27 participants. (B) Study participants’ sinus microbial communities were categorized as being relative stable or unstable over time. Shown are results from histogram binning of study participants based on the standard deviation (SD) of each of their study visit’s Manhattan distances from the first microbiota sample sequenced. The dotted line indicates the median SD across all participants. *n* = 15 study participants with 3 or more visits. (C) Example of a study participant (patient 41), categorized as relatively stable based on the results in panel B, whose sinus microbiota was consistently dominated by P. aeruginosa over time. Taxonomic bar plots depict the relative abundance of *Pseudomonadaceae* (P. aeruginosa [peach]). The overlaid line plotted on the right *y* axis is the Manhattan distance of each sample from their first microbiota sample. (D) Example of a study participant (patient 33), categorized as unstable based on the results in panel B, who exhibited switching between P. aeruginosa and Staphylococcus species dominance over time. Taxon bar plots depict the relative abundance of *Pseudomonadaceae* (P. aeruginosa [peach]), Staphylococcus spp. (red), *Corynebacterium* spp. (purple), and other low-abundance taxa. The overlaid line plotted on the right *y* axis is the Manhattan distance of each sample from its first microbiota sample. (E) FISH images of CF CRS microbial communities from explanted obstructive sinus debris sampled by endoscope from two study participants. In the left panel (patient 45), the S. aureus probe is red, the eubacterial (universal) probe is green, and Hoechst stain is blue (mostly host cell nuclei). In the right panel (patient 33), P. aeruginosa is red, the eubacterial (universal) probe is green, and Hoechst stain is blue. Scale bars = 50 μm. The macroscopic image at the top left of each FISH image depicts the mucopurulent sinus sample prior to processing for microscopy.

Sinus microbial community diversity in our cohort was low, with the median Shannon diversity from all participants being 0.35 (interquartile range [IQR], 0.11 to 0.62) and Simpson diversity of 0.18 (IQR, 0.04 to 0.35) (Fig. 1A). The median evenness was 0.15 (IQR, 0.04 to 0.25), suggesting communities were dominated by a subset of the taxa present. The Tail statistic (τ), a rank-based diversity measure that is more sensitive to changes in low-abundance taxa, was also examined (17). The median τ was 0.46 (IQR, 0.27 to 0.66), but it had a fairly large range (from 0.04 to 5.6), indicating that diverse low-abundance taxa are present in the sinuses of some, but not all, study participants (see Table S1 in the supplemental material). We detected a total of 302 genera (Table S2).

While diversity indices of the sequenced sinus microbiotas were low, microbial community composition was unstable for many study participants (Fig. 1B). For individuals that contributed at least 3 longitudinal microbiota samples, we quantified this instability based on the standard deviation (SD) of the microbiota distance (Manhattan distance) at later time points relative to the first time point. We grouped individuals based on the median value for the study cohort, with the eight individuals whose values fell below the median classified as relatively stable (for example, the individual whose taxonomic bar plots are shown in Fig. 1C) and seven as relatively unstable (for example, in Fig. 1D). The average length of time from enrollment to the final study visit did not differ between groups; however, individuals with unstable microbiotas tended to have more study visits than those whose communities were stable. This highlights the importance of frequent longitudinal sampling to detecting microbiota instability.

The most common bacterial taxa were Staphylococcus spp. and *Pseudomonadaceae* (Table S2), and the relative abundances of these two taxa varied over time in several study participants from whom we collected longitudinal samples (Fig. 1D and Fig. S1). Based on Sanger sequencing of the 16S rRNA gene following bacterial culture, the *Pseudomonadaceae* taxon represents Pseudomonas aeruginosa and is referred to as such here. We then examined the biogeography of P. aeruginosa and/or Staphylococcus aureus in these communities, using fluorescent *in situ* hybridization (FISH) on explanted obstructive sinus material that was surgically debrided as part of routine clinical care. We found that both P. aeruginosa and S. aureus reside as small aggregated communities in close association with host cells in the sinuses (Fig. 1E). Eubacterial labeling did not fully overlap species-specific probes, suggesting other unidentified species were present in mixed-species aggregates. Overall, these results suggest that while CF CRS microbes can reside in small, sparse aggregates where diversity is low, the aggregates can contain mixed species in proximity with each other and with host cells. Furthermore, the overall taxonomic composition of sinus communities can be rather unstable, especially in individuals coinfected by P. aeruginosa and Staphylococcus spp.

While P. aeruginosa and Staphylococcus spp. were abundant in many study participants, other microbes were stably present as well. Regarding patterns of co-occurrence among microbes, we identified positive correlations between the presence of *Corynebacterium* spp. and *Dolosigranulum* spp., whereas P. aeruginosa and *Burkholderia* spp. were negatively correlated (Fig. S2). In addition to taxa recognized as members of the nasal, sinus, or oral microbiotas of healthy adults, we identified bacteria known to be present in potable water and capable of causing opportunistic infections in susceptible populations (e.g., *Sphingomonas* spp. in 11 out of 27 participants, *Bradyrhizobium* spp. in 10, *Methylobacterium* spp. in 9, and *Delftia* spp. in 6) (Table S2) (18). The composition of environmental and reagent control samples processed and sequenced alongside our study specimens was distinct from that of clinical specimens (by permutational multivariate analysis of variance [PERMANOVA], *R*^2^ = 0.0822 and *P* < 0.0001), and the controls had significantly lower read counts than the study samples (by *t* test, *P* < 0.0001). These controls suggest that the potable water taxa that were identified in the clinical specimens were not due to contamination during sample collection, processing, or sequencing. Overall, these findings demonstrate that while most sinus communities were dominated by P. aeruginosa and/or Staphylococcus spp., a variety of other taxa were also detected. The presence of bacteria frequently reported to be present in potable water suggests a potential exposure route of the sinuses to opportunistically pathogenic microbes that contribute to diversity of low-abundance taxa.

### *Pseudomonas* spp. and *Staphylococcus* spp. drive community structure and low diversity.

**FIG 2:**
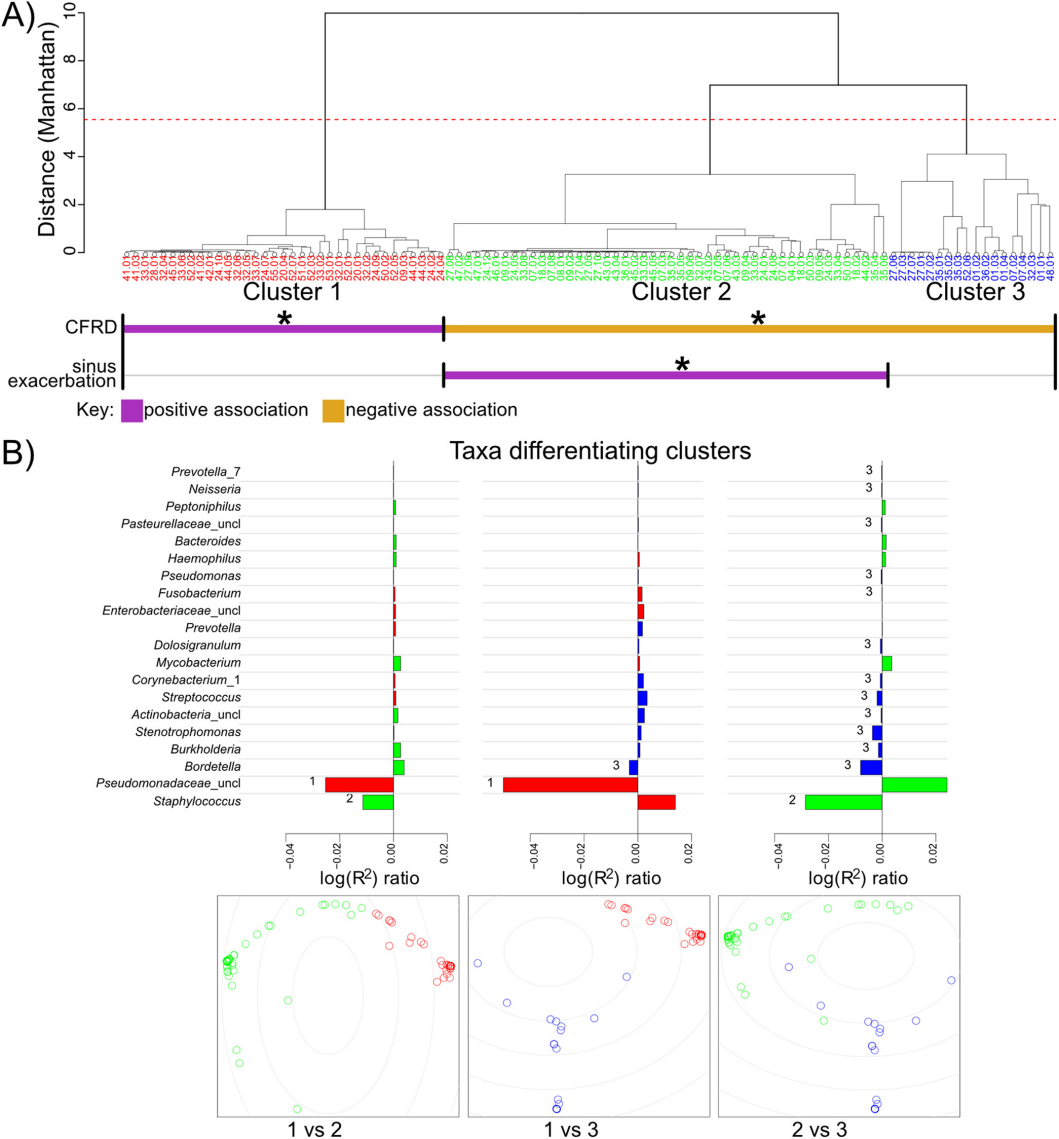
CF sinus microbial community structure is driven by relative abundance of Staphylococcus spp. or P. aeruginosa. (A) Dendrogram depicting individual patient samples hierarchically clustered using Ward’s minimum variance method on the intersample Manhattan distance. Individual microbiota samples are colored based on their cluster: 1 is red, 2 is green, and 3 is blue. Covariates or sinus disease outcome measures that significantly correlated with cluster membership by multinomial linear regression are summarized below the clusters. CFRD is positively associated with cluster 1 and negatively associated with clusters 2 and 3. Sinus exacerbation is positively associated with cluster 2. Cluster 1 is driven by P. aeruginosa, and cluster 2 is driven by *Staphyloccus* spp. (B) Which and to what extent each taxon drives the differences between clusters were calculated by comparing the coefficient of determination (*R*^2^) for a reduced model (without a taxon of interest) against that for a full model (with all taxa included) with the ratio of reduced *R*^2^ to full *R*^2^. If excluding a taxon (evaluated with the reduced model) reduces the separation between two clusters relative to keeping it (evaluated with the full model), then it was an important taxon to the cluster, and the *R*^2^ ratio would be <1. Values plotted are log_10_(reduced *R*^2^/full *R*^2^), with negative values indicating the most influential taxa separating the two clusters compared. The bottommost plots are classical multidimensional scaling (MDS) plots depicting the separation of individual samples in the indicated clusters.

**FIG 3:**
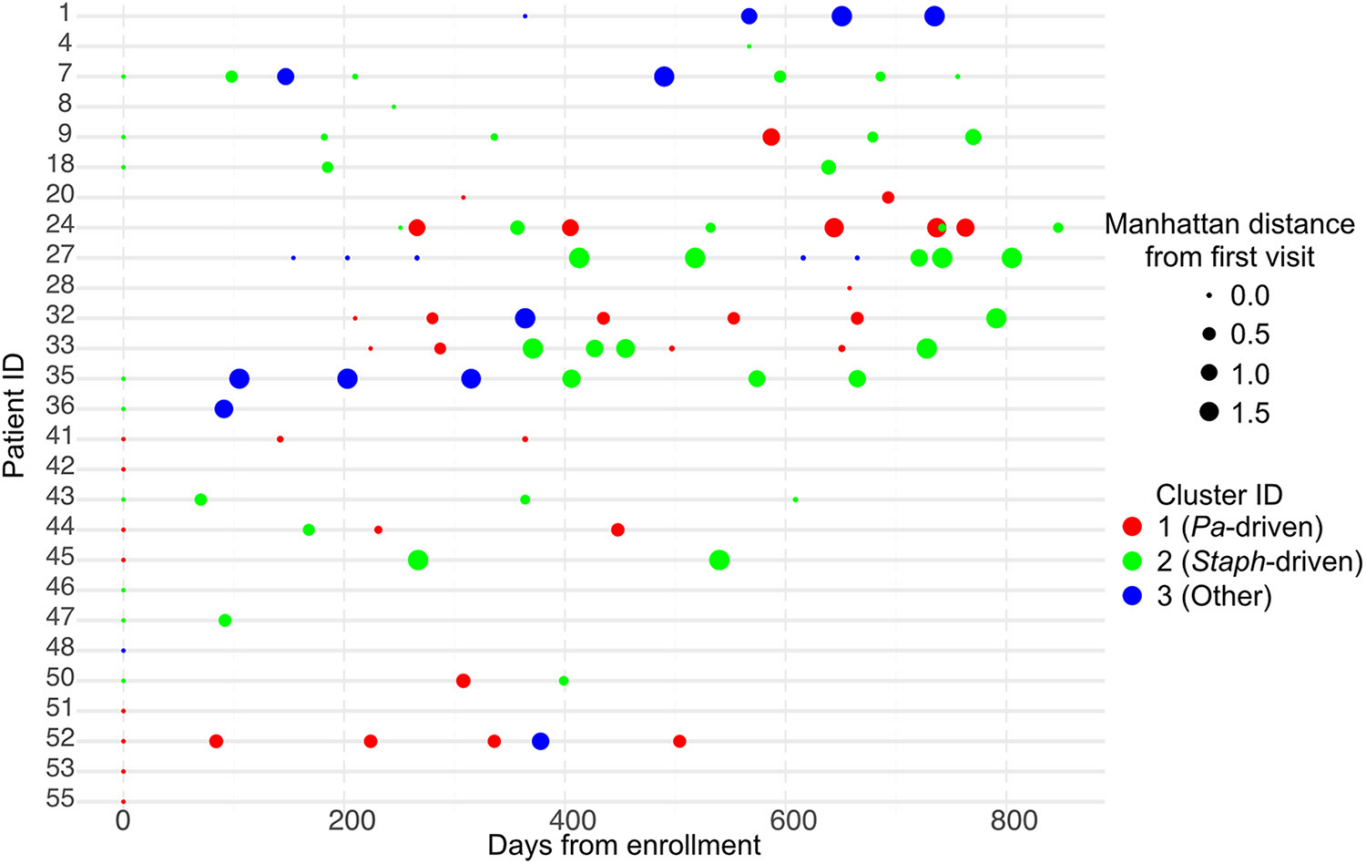
Individuals’ sinus microbiota structure switches between being driven by P. aeruginosa, Staphylococcus spp., or other taxa over time. Twelve of 18 individuals with longitudinal microbiota samples switch cluster membership over time. Cluster membership is colored as in Fig. 2. Cluster 1 (the P. aeruginosa-driven cluster) is red, cluster 2 (the Staphylococcus species-driven cluster) is green, and cluster 3 (the cluster driven by other taxa) is blue. The size of each dot is proportional to the Manhattan distance at each time point from the first sequenced sample.

To further interrogate the drivers of sinus microbial community structure in CF, we performed a hierarchical cluster analysis (Fig. 2A). We found that individual sinus samples grouped into three clusters (Fig. S3A), with separation of the two largest clusters driven by the relative abundances of Pseudomonas spp. and Staphylococcus spp. and the third cluster driven by the relative abundance of a mix of other taxa that were less prevalent in our cohort (Fig. 2B). Similarly, these two dominant taxa were also found to unify different groups, with Pseudomonas spp. unifying cluster 1, whereas Staphylococcus spp. unified cluster 2 (Fig. S3B). Consistent with the instability observed in Fig. 1, study participants did not tend to belong to solely one cluster. Instead, most participants’ sinus microbiotas frequently switched between clusters over time, depending on the relative abundance of Pseudomonas spp. and/or Staphylococcus spp. at that time point (Fig. 3).

**FIG 4:**
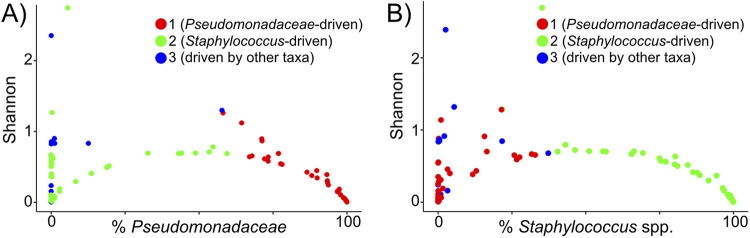
Relative abundance of Staphylococcus spp. or P. aeruginosa almost exclusively drives low microbial diversity and community structure. (A) Microbial community diversity (Shannon) is reduced as the relative abundance of P. aeruginosa (cluster 1 samples in red and *x* axis) or Staphylococcus spp. (cluster 2 samples in green) begin to dominate. Individual samples that were clustered in Fig. 2A were plotted by the relative abundance of P. aeruginosa (*x* axis) and their Shannon diversity index (*y* axis) and then were colored by their cluster membership. Samples in cluster 3 (blue) tended to have higher Shannon diversity values than those in cluster 1 or 2. (B) Shannon diversity decreases as the relative abundance of Staphylococcus spp. increases. The cluster membership of each sample is indicated by colors as in panel A. Samples with the highest relative abundance of Staphylococcus spp. belong to cluster 2 (green).

Using the stability classification from Fig. 1B, six of the seven individuals with unstable microbiotas switched between clusters in Fig. 3, whereas five of the eight individuals with a stable sinus microbiota exhibited cluster switching. The individual from the unstable group who did not switch clusters (patient 1 in Fig. S1) stayed within cluster 3 (a cluster driven by a mix of taxa other than Pseudomonas spp. or Staphylococcus spp.), but their sinus microbiota transitioned from Streptococcus to *Burkholderia* species dominance, which is why they were grouped among the “unstable” population despite not switching clusters. In contrast, most of the relatively stable individuals who switched clusters were coinfected with P. aeruginosa and Staphylococcus spp. Their cluster switching was due to changes in relative abundance of these two taxa that drove them between clusters 1 (Pseudomonas-driven cluster) and 2 (Staphylococcus-driven cluster), yet did not lead to a high enough variability in Manhattan distances to categorize them as unstable in Fig. 1B because they remained coinfected. Furthermore, the increased relative abundance of P. aeruginosa or Staphylococcus spp. tracked with decreasing Shannon diversity (Fig. 4A and B), suggesting that the low sinus community diversity is attributable to dominance by these two taxa. This reduction in Shannon diversity as the relative abundance of Pseudomonas spp. or Staphylococcus spp. increases was most apparent in samples from clusters 1 and 2.

Examining clinical correlates of microbiota cluster membership, we found CFRD was positively associated with cluster 1 membership (Pseudomonas-driven cluster) and negatively associated with clusters 2 and 3 (clusters driven by Staphylococcus spp. or other taxa) (Fig. 2A). In agreement with this clustering analysis, we found that people with CFRD had a higher relative abundance of Pseudomonas spp. than those without CFRD (regression coefficient, 45.35; *P* < 0.01) (Table 2). Finally, sinus exacerbation was positively associated with cluster 2 membership (driven by Staphylococcus spp.). These results demonstrate how further clustering sinus microbiotas of people coinfected by P. aeruginosa and Staphylococcus spp. based on drivers of community structure can reveal relationships with comorbidities (CFRD) or disease status (sinus exacerbation).

**TABLE 2:** Associations of taxa with CFRD^*a*^

Taxon	Coefficient	Unadjusted *P* value
P. aeruginosa	45.35	<0.01
Streptococcus spp.	−46.82	<0.001
*Fusobacterium* spp.	31.30	<0.001

a Shown are the linear regression coefficients describing a positive association of CF-related diabetes (CFRD) with *P. aeruginosa* and *Fusobacterium* spp. and a negative association with *Streptococcus* spp. The top 15 most abundant taxa were tested for associations with CFRD. The following covariates and clinical variables were included in the model: patient ID, age, CFRD, current topical antibiotic use, FEV_1_, SNOT-22 and mLK scores, and whether a patient was experiencing a sinus or pulmonary exacerbation.

### Sinus microbiotas are highly individualized, but may share a signature during sinus exacerbation.

We used permutational multivariate analysis of variance (PERMANOVA) to determine whether community-level differences in sinus microbiotas associated with topical antibiotic usage or current respiratory disease status (sinus or pulmonary exacerbation status at the time of the clinic visit). We found that the variation in community composition across the cohort was largely explained by the study participant who contributed the sample (by PERMANOVA, *R*^2^ = 0.51 and *P* < 0.001) (Table S3), suggesting that sinus microbiotas are highly individualized. However, whether a participant was experiencing a sinus exacerbation at the time of the study visit also explained a small amount of variability (by PERMANOVA, *R*^2^ = 0.016 and *P* = 0.066) (Table S3). This suggests that a common signature of disturbance in the CF CRS microbiota may occur during sinus exacerbations.

### Worsened sinus disease is associated with reduced microbial community diversity and changes in relative abundance of *Gammaproteobacteria*.

**FIG 5:**
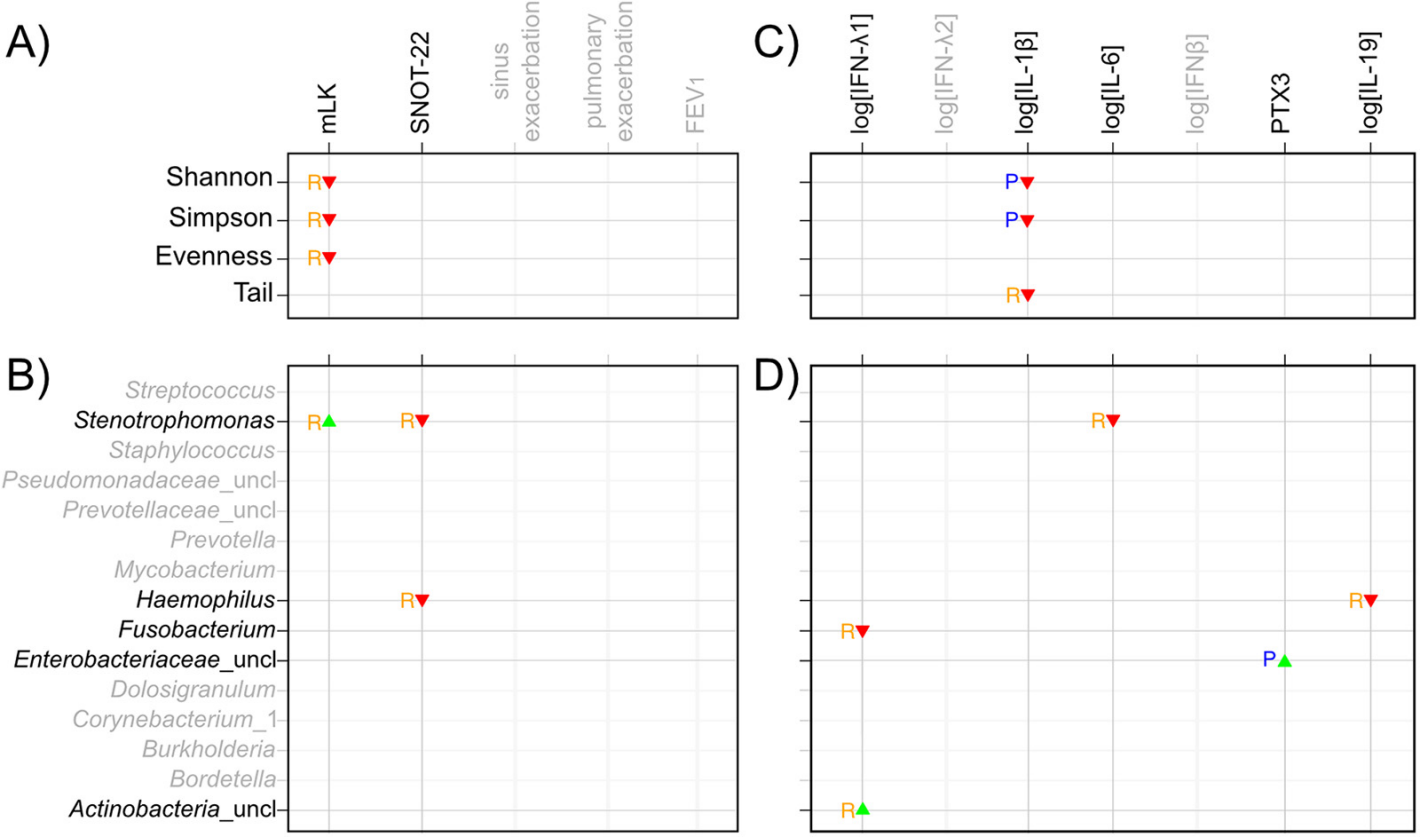
Relationship of microbial community diversity and individual taxa with respiratory disease outcomes and pro- or anti-inflammatory cytokines. A summary matrix depicts statistically significant relationships between clinical outcome variables (A and B) or cytokines (C and D) with alpha diversity (A and C) or the top 15 taxa (B and D). The blue P’s represent the associations when the alpha diversity indices or taxa best predict (“P”) the cytokine values or clinical variables. In contrast, the orange R’s depict when the clinical variables or cytokines predict the alpha diversity indices or taxa with greater statistical significance (i.e., when the alpha diversity indices or taxa respond [“R”] to the cytokine values or clinical variables). The green upward-pointing triangles represent positive associations between diversity or taxa and clinical variables or cytokines, whereas the red downward-pointing triangles represent negative associations based on the coefficients of their association. To compute the associations, four pairs of linear regression models were fit. In panel A, (i) diversity indices = covariates + clinical variables and (ii) clinical variables = covariates + diversity indices. In panel B, (iii) taxa = covariates + clinical variables and (iv) clinical variables = covariates + taxa. In panel C, (v) diversity indices = covariates + cytokines and (vi) cytokines = covariates + diversity indices. In panel D, (vii) taxa = covariates + cytokines and (viii) cytokines = covariates + taxa. Taxa are represented as additive log ratio-transformed relative abundances. Cytokine concentrations were log transformed, except for pentraxin 3 (PTX3), which was sufficiently normally distributed. Covariates included patient ID, age, sex, BMI, CFTR mutation, CFRD status, and topical antibiotic usage. Associations included in the predictor/response matrix required at least one of the associations from the models to have an estimated coefficient *P* value of <0.025. The coefficients for relationships depicted in each panel are as follows: (A) Shannon responds to mLK, −0.138, Simpson responds to mLK, −0.169, and evenness responds to mLK, −0.138; (B) *Stenotrophomonas* responds to mLK, 0.287, *Stenotrophomonas* responds to SNOT-22, −0.0691, and Haemophilus responds to SNOT-22, −0.0483; (C) Shannon predicts log[IL-1β], −2.64, Simpson predicts log[IL-1β], −4.2, and Tail responds to log[IL-1β], −0.233; (D) *Stenotrophomonas* responds to log[IL-6], −1.09, Haemophilus responds to log[IL-19], −0.287, *Fusobacterium* responds to log[IFN-λ1], −0.342, *Enterobacteriaceae*_uncl predicts PTX3, 341, and *Actinobacteria*_uncl responds to log[IFN-λ1], 0.85.

Because the PERMANOVA suggested a distinct microbial community associated with sinus exacerbation and because reduced sputum microbiota diversity is correlated with worse lung function in CF (19–21), we next asked whether a similar relationship between microbial community diversity and disease occurs in the sinuses during CF CRS. We used a predictor versus responder statistical test to compare models that use diversity indices or specific taxa as predictors of respiratory disease outcomes against models that use the same indices or taxa as responders to disease outcomes (22). In this analysis, we report that diversity indices or taxa “respond” to disease outcomes when disease outcomes more strongly predict diversity indices or taxa than vice versa, but it is important to note that the biological basis of these statistical associations was not investigated experimentally. We found that microbial community diversity (Shannon and Simpson) and evenness decreased in response to increasing mLK score (Fig. 5A), suggesting that a more diverse sinus microbiota, not dominated by one or very few taxa, is associated with less severe sinus disease.

We next examined whether any of the top 15 most abundant taxa were associated with the same disease outcomes in the predictor versus responder statistical test (Fig. 5B). We found a positive relationship between *Stenotrophomonas* spp. and physician-scored sinus disease (mLK scores ranged from 4 to 16 across study participants’ visits in our study), in which the relative abundance of *Stenotrophomonas* spp. increases in response to increasing mLK score. In contrast, the relative abundance of two taxa, *Stenotrophomonas* spp. and Haemophilus spp., decreased in response to worse symptomatic sinus disease (higher SNOT-22 scores). The relative abundance of *Stenotrophomonas* spp. ranged from 0% in some study participants to over 80% in patient 35’s fourth visit, and the relative abundance of Haemophilus spp. ranged from 0 up to 17.6% in patient 18’s second visit (Fig. S1). We did not detect statistically significant relationships between these diversity indices or taxa and exacerbation (sinus or pulmonary) or FEV_1_. Together, these data suggest relationships between microbial community diversity and, specifically, two *Gammaproteobacteria* (*Stenotrophomonas* spp. and Haemophilus spp.) with upper respiratory disease severity. In the case of the opportunistic pathogen *Stenotrophomonas*, the relationship differs, depending on whether the outcome of interest is patient- or physician-scored sinus disease.

### Low sinus community diversity and the relative abundance of several taxa are associated with cytokine changes.

Respiratory disease progression in both CRS and CF is thought to be caused by cycles of infection and inflammation. Therefore, we sought to identify relationships between the sinus inflammatory environment and community diversity, as well as specific taxa. Using the same predictor versus responder approach, we identified a negative relationship between the levels of the proinflammatory cytokine interleukin-1β (IL-1β) and three microbiota diversity indices. Higher Shannon or Simpson diversity predicted decreased IL-1β levels (coefficient [*P* value], Shannon/log[IL-1β], −2.64 [0.0222]; Simpson/log[IL-1β], −4.2 [0.0223]) (Fig. 5C). Similarly, the diversity of low-abundance taxa (Tail statistic) decreased in response to higher levels of IL-1β (−0.233 [0.00058]) (Fig. 5C). Furthermore, the relative abundances of several taxa exhibited relationships with the host cytokine response (Fig. 5D). The relative abundances of some taxa decreased in response to increasing levels of cytokines. *Stenotrophomonas* spp. decreased in response to increased levels of proinflammatory IL-6 (−1.09 [0.0186], Haemophilus spp. decreased in response to anti-inflammatory IL-19 (−0.287 [0.000637], and *Fusobacterium* spp. decreased in response to proinflammatory lambda 1 interferon (IFN-λ1 [also known as IL-29]) (−0.342 [0.000653]). The relative abundances of other taxa exhibited positive correlations with some cytokines. Increased relative abundance of unclassified taxa within the family *Enterobacteriaceae* predicted increased levels of the proinflammatory cytokine pentraxin 3 (341.0 [0.0213]), whereas the relative abundance of unclassified taxa within the phylum *Actinobacteria* increased in response to increasing concentrations of IFN-λ1 (0.85 [0.0211]). These findings suggest that sinus inflammation is highest when microbial community diversity is low.

## DISCUSSION

The unified airway hypothesis suggests that management of upper airway disease (e.g., CRS) could benefit the lower airways. However, the understudied nature of CF sinus disease means evidence-based recommendations for management of CF CRS are currently lacking (23). In the present study, we sought to determine how sinus microbial community structure and composition in adults with CF CRS change across disease and inflammatory states over time. We found that while communities lacked diversity and tended to be dominated by P. aeruginosa or Staphylococcus spp., many were noticeably unstable over time. Our work also revealed a link between Staphylococcus species dominance and sinus exacerbation, as well as potential interplay among P. aeruginosa dominance in the sinuses, CFRD, and reduced lung function. Proinflammatory cytokine responses were associated with decreased sinus microbial community diversity and changes in relative abundance of several taxa. Together, these findings shed light on potential host-microbe interactions occurring in the sinuses during CF CRS and have implications for the design of future studies aimed at linking CF CRS phenotypes to disease outcomes. Our work also provides support for investigation of therapeutic strategies targeting IL-1β for CF sinus disease.

### Implications of sinus microbiota instability in people with CF CRS.

We were surprised to observe instability in CF CRS microbial communities over time, even among individuals whose communities were dominated by P. aeruginosa or Staphylococcus spp. Such instability has been implicated with worse lung function in a meta-analysis of CF sputum microbiotas from several cohorts (24) and, similarly, in non-CF CRS and diseases involving other microbiome-mucosal interfaces, such as in the gut (25, 26). Whether and how the sinus community instability observed in our study is linked to overall respiratory disease progression warrants future investigation, but some parallels can be drawn to existing ecological models of CF lung disease. For example, the climax-attack model of CF pulmonary exacerbation is rooted in the concept of unstable respiratory communities. In this model, the presence/absence or relative abundance of taxa associated with an “attack” (community associated with exacerbation, inflammation, and tissue damage) or “climax” (community dominating during periods of clinical stability) community cycles over time, leading to repeated periods of inflammation, pulmonary exacerbation, and progressive tissue damage (27). It is possible that the most unstable communities in our study could be cycling between attack and climax communities, similarly driving sinus disease progression. These communities may also be shifting in response to changing antimicrobial treatments.

Another explanation for the observed sinus microbiota instability could be related to the infection site biogeography, specifically the size and structure of bacterial aggregates present in the paranasal sinuses. Using an advanced imaging technique called MiPACT-HCR (microbial identification after passive clarity technique and hybridization chair reaction) (28), we recently discovered that the size and structure of P. aeruginosa populations varied drastically in adults with CF CRS who were also members of the present cohort (10). These variations in population sizes impacted ongoing genome evolution and adaptation. In the present study, we imaged two additional sinus microbial populations (including one that contained S. aureus) and observed small, sparse aggregates of bacteria in both participants. Therefore, another explanation for the apparent instability could be due to community compositional differences among isolated aggregates in a structured sinus environment where different taxa may occupy distinct niches. In this case, sampling small, sparse, taxonomically heterogeneous microbial populations could result in apparently unstable populations by random chance, whereas sampling large, taxonomically well-mixed populations could appear to be more stable over time.

Notably, our examination of microbial community instability is limited by the fact that our microbiota analyses are compositional and not quantitative. Future studies examining the apparent instability of CF CRS microbiotas should endeavor to quantify changes in the absolute abundance of bacteria present at the sampling site (29, 30). A future longitudinal study would also benefit from more frequent sample collection with regularly spaced intervals of time between samples to better assess microbial community stability or instability. More broadly speaking, CF respiratory disease is chronic and progressive, with cycles of mucus obstruction, infection, and inflammation driving worsening of respiratory health over an individual’s lifetime (31). Predictors of exacerbation or biomarkers of disease progression would greatly inform clinical care, but are currently lacking. The instability identified in our study highlights a potential hurdle for cross-sectional studies that aim to link relative abundance of taxa to outcome measures that develop over longer periods of time (rather than outcomes of acute phenomena); this limitation should be taken into consideration for future study design.

### Comparison to prior studies of CF respiratory microbial community structure.

Our findings share similarities and build upon prior microbiota studies of CF CRS and CF sputum examining diversity and taxonomic drivers of community structure. Consistent with previous adult CF CRS and sputum studies, microbial community diversity was low (4, 5, 19, 32–35), and communities were frequently dominated by P. aeruginosa or staphylococci (4–6, 35–38). Lucas et al. recently showed that low diversity was associated with dominance by Pseudomonas spp. and was not significantly associated with the clinical factors examined (36), whereas we found that both P. aeruginosa and Staphylococcus spp. can drive low community diversity and that low diversity is associated with worse sinus disease as measured by mLK score. Additionally, a recent longitudinal study of four adults with CF CRS reported marked stability in sinus communities over time (38), whereas approximately half of our cohort exhibited instability. These inconsistencies between studies could be due to differences in study design (e.g., cohort sizes or cross-sectional versus longitudinal), methodological differences in sequencing or analysis approaches, or potentially differences in the cohorts themselves, all of which were recruited at single, distinct study centers. While our 16S amplicon sequencing approach did not detect large taxonomic changes in the group of individuals classified as relatively stable, it is important to note a limitation of this approach. We are unable to determine whether the metabolic activity, for example, of the consortia of stable taxa shifts over time in ways that would achieve functionally similar changes to the taxonomic shifts in the relatively “unstable” individuals, as has previously been described in the oral cavity (39). On the other hand, taxonomic drivers of the three microbial community clusters identified in our study share similarities with the five clusters recently identified by Hampton et al. in a meta-analysis of CF sputum microbiomes from multiple adult cohorts (24). We observed a link between cluster membership and sinus disease exacerbation, whereas Hampton et al. linked sputum cluster membership to variability in lung function. Specifically, we found that sinus exacerbation was associated with having a microbial community driven by Staphylococcus spp. (cluster 2 membership). This result is consistent with a clinical study of nasal lavage samples from CF adults, which found that colonization of the upper airway by S. aureus (as determined by clinical lab culture) was associated with increased levels of several proinflammatory cytokines, whereas no association was detected for P. aeruginosa (40). It is also consistent with the prior publication from our research group on clinical indicators of sinus disease, which found a link between sinus S. aureus colonization (as determined by clinical lab culture) and worsening sinus disease (12). Together, these studies reveal that beyond resembling each other taxonomically, CF URT and LRT microbial communities also share similar drivers of their structure, some of which can be linked to sinus disease.

### Links between sinus microbial community diversity, inflammation, and upper respiratory disease severity.

CRS is an inflammatory disease, and we found several examples of how cytokine signaling in the sinuses could shape CF CRS microbial communities. Multiple lines of evidence link elevated levels of the proinflammatory cytokine IL-1β to sinus disease. Polymorphisms in the IL-1 receptor antagonist gene (*IL1RN*) are associated with CRS, and in CF, elevated IL-1β is associated with the presence of nasal polyps (41–44). We found that lower microbial community diversity was associated with higher levels of IL-1β and with worse endoscopic appearance of the sinuses (mLK score). Our findings suggest IL-1β signaling in the sinuses as a therapeutic target to control sinus inflammation and potentially restore microbial community diversity. Considering a recombinant IL-1β receptor antagonist is already available to treat rheumatoid arthritis and other inflammatory diseases, this finding warrants further investigation (45). These cytokine interactions suggest ways that the inflammatory environment of the sinuses could reinforce a lack of diversity and dominance by more abundant taxa, including P. aeruginosa and Staphylococcus spp.

### Relationships of specific taxa with each other and the host environment, including during CFRD.

The CF lung environment displays a high degree of interplay between host-microbe and microbe-microbe interactions that impact bacterial behaviors such as expression of virulence factors and response to antimicrobials (46). The strongest signature of microbe-microbe co-occurrence in our study was a positive correlation between *Corynebacterium* spp. and *Dolosigranulum* spp., which has previously been observed in the upper respiratory tract of healthy individuals and associated with relative stability of the microbiota (47, 48). We interpret these findings to suggest that CF CRS sinuses continue to harbor a commensal subpopulation displaying interactions seen outside the context of CF (47, 49, 50). We also observed a negative correlation between the relative abundance of P. aeruginosa and *Burkholderia* spp. A type VI secretion-mediated mechanism of antagonism between these two organisms was recently demonstrated to evolve among CF sputum isolates, further suggesting similarities between polymicrobial interactions in the URT and LRT (51).

Finally, we observed a correlation between CFRD and reduced lung function, as was previously reported (12). Adding to this finding, here we identified a relationship between CFRD and sinus communities driven by P. aeruginosa (many of which also contained Staphylococcus spp.). CFRD increases in prevalence as individuals age (52), yet age was not statistically associated with the P. aeruginosa-driven cluster in our cohort. How the sinus environment during CFRD might promote P. aeruginosa infection independent of potential confounders like age and worse lung disease is a future direction for mechanistic studies that follow up on this observation. Garnett et al. used a CF bronchial epithelial cell culture model of hyperglycemia to link elevated paracellular glucose flux, lactate secretion, and increased airway acidification to increased P. aeruginosa growth (53, 54). Furthermore, Limoli et al. reported that LRT coinfection by P. aeruginosa and S. aureus is common among people with CFRD (55). These parallels between our findings and previous reports from studies of the LRT in CF further underscore the relevance of the URT to overall CF respiratory disease dynamics. A limitation of our study is that we do not have paired information about the microbiota of the LRT. To further examine CF sinus disease in the context of the unified airway hypothesis, comparison of microbiotas from paired sinus and sputum samples collected longitudinally is needed to examine how these two populations relate to each other and change over time, including during disease exacerbations.

### Summary perspective: treating CF sinus disease in the new era of highly effective modulator therapies.

In the United States, the CF Foundation recently published guidelines for the management of CF CRS (14). Our study advances the growing body of literature establishing the sinuses as an important site of chronic infection along the respiratory tract of people with CF. The CF community has entered the era of highly effective CF transmembrane conductance regulator (CFTR) modulator therapy (HEMT), in which widespread use of HEMT is changing CF disease in unprecedented ways that will require complementary changes in how CF is managed for many people (56). Initiation of HEMT early in life may delay respiratory disease progression. Prevention of sinus infection and management of sinus disease could offer an additional opportunity to prevent or delay LRT disease, as well as to relieve the symptomatic quality of life burden for people with CF. With respect to infection prevention, we identified taxa associated with potable water among CF sinus microbial communities. Potable water has long been hypothesized to be a source of P. aeruginosa that infects the CF respiratory tract (57), but evidence from molecular epidemiological studies that seek to match P. aeruginosa from the homes of people with CF to their infecting isolates is mixed (58–62). Our study suggests a route of exposure of the sinuses to opportunistic pathogens from potable water that could be intervened upon to prevent or delay infection of the sinuses. As therapeutic options for CF are expanded and life expectancy extended, a comprehensive understanding of the ecological and evolutionary drivers of CF sinus disease holds promise for more rational interventions and treatment of chronic respiratory infections in people with CF.

## MATERIALS AND METHODS

### Study design, participants, sinus sampling, and clinical evaluation.

We performed a prospective, longitudinal study of 33 CF adults with symptomatic CRS and prior functional endoscopic sinus surgery (FESS) following an IRB-approved protocol (STUDY19100149) between February 2015 and August 2017 (12). Participants were treated in a CF-focused otolaryngology clinic at the University of Pittsburgh. During quarterly clinic visits and unscheduled clinic visits, at least two sinus swabs were collected endoscopically for 16S rRNA gene amplicon sequencing (dry flock swab; Puritan Medical Products, Guilford, ME) and bacterial culturing (flocked swab with liquid Amies medium; Copan Diagnostics, Inc., Murrieta, CA). Samples were collected from the frontal, maxillary, or ethmoid sinuses (see Table S4 in the supplemental material). The swab for bacterial culturing was stored on wet ice and cultured within 4 h of sampling. Sinus wash was collected for cytokine analysis by flushing 5 mL of sterile saline into the sinus cavity and collecting the sample endoscopically with a sterile trap. Sinus washes and dry swabs were stored at −80°C. Of the 33 participants enrolled in the study and for whom sinus samples had been collected, we sequenced microbiota samples on at least two different study visits from 18 people (i.e., longitudinal samples) and sequenced a single cross-sectional sample from nine people for a total cohort size of 27 people (Table 1). Patient demographics, clinical characteristics, including the criteria for disease outcome variables “sinus exacerbation” and “pulmonary exacerbation,” and medication use were previously described for the full 33-person cohort, and the same definitions were used in the present study (12). Briefly, a sinus disease exacerbation was defined as an unscheduled visit to the sinus clinic (i.e., a visit that was outside regular study visits) and/or if the study participant reported an acute increase in symptom severity. A pulmonary exacerbation occurred if at least two of the following three occurred within 4 weeks of a study visit: (i) a greater than 10% drop in percentage of predicted FEV_1_, (ii) institution of a new course of systemic antibiotics by the pulmonary team, and (iii) documentation by the treating pulmonologist that the study participant was experiencing a pulmonary exacerbation in the medical record. Table S4 contains the study’s clinical metadata, and Table S5 is a codebook describing each variable. The topical antibiotic usage data included in the metadata reflect topical antibiotics prescribed in the CF Sinus Clinic, whereas oral and intravenous (i.v.) antibiotic usage was determined retrospectively from chart reviews.

### DNA extraction and 16S rRNA gene amplicon sequencing.

DNA extraction was performed using the Qiagen DNeasy Powersoil kit (catalog no. 12888; Qiagen, Germantown, MD) and processed following the manufacturer’s protocol. Reagent blanks were included as negative controls, and cells from a microbial community of known composition were included as positive controls (ZymoBIOMICS microbial community standard; Zymo Research, Irvine, CA). The V4 region of the 16S rRNA gene was amplified from ~5 to 10 ng of extracted DNA in 25-μL reaction mixtures using Q5 HS high-fidelity polymerase (New England BioLabs, Ipswich, MA) with inline barcoded primers designed as previously described (63). The following V4-specific primers were used: 515f (5′-GTGCCAGCMGCCGCGGTAA-3′) and 806r (5′-GGACTACHVGGGTWTCTAAT-3′). The cycle conditions were 98°C for 30 s, followed by 30 cycles of 98°C for 10 s, 57°C for 30 s, and 72°C for 30 s, and then a final extension step of 72°C for 2 min. We used two-sided AMPure XP bead purification at 0.8:1 (left side) and 0.61:1 (right side) ratios to remove small and large fragments, respectively. Eluted DNA was quantified on a Qubit fluorimeter (Life Technologies, Grand Island, NY). Samples were pooled on ice by combining 40 ng of each purified band. For negative controls and poorly performing samples, 20 μL of each sample was used. The sample pool was purified with the MinElute PCR purification kit (Qiagen, Germantown, MD). The final sample pool underwent two more purifications: AMPure XP beads at a ratio of 0.8:1 to remove primer dimers and a final cleanup using the Purelink PCR purification kit (catalog no. K310001; Life Technologies, Grand Island, NY). The purified pool was quantified in triplicate with a Qubit fluorimeter prior to sequencing.

Amplicons of the V4 region were sequenced on a MiSeq (Illumina, San Diego, CA) using paired-end 2 × 250-bp reads, deconvolved, and quality checked by DUST low-complexity filtering, quality value (QV) trimming, and trimming of primers used for 16S rRNA gene amplification by the University of Pittsburgh’s Center for Medicine and the Microbiome (CMM) using the scripts fastq_quality_trimmer and fastq_quality_filter from Hannon's Cold Spring Harbor Laboratory's FASTAX-Toolkit (http://hannonlab.cshl.edu/fastx_toolkit/). Reads were trimmed until the QV was 30 or higher. Trimmed reads shorter than 75 bp or those with less than 95% of the bases above a QV of 30 were discarded. Forward and reverse paired reads were merged with a minimum required overlap of 25 bp, proportion overlap mismatch of >0.2 bp, maximum *N*'s allowed of 4, and a read length minimum of 125 bp. Reads were taxonomically classified with Mothur version 1.39.1 (16), using Ribosomal Database Project (RDP v123) reference sequences (64). Environmental controls and extraction kit controls, along with Escherichia coli and mock community (ZymoBIOMICS microbial community DNA standard) positive controls, were sequenced alongside clinical specimens to monitor for contamination and technical performance during the extraction and sequencing process.

### Verification of the *Pseudomonadaceae*_uncl taxon as *P. aeruginosa*.

For every study visit, a sinus swab was streaked onto Pseudomonas isolation agar (PIA) and incubated at 37°C for 48 h. Genomic DNA from a representative isolate or isolates for each study participant was extracted using a Qiagen DNeasy blood and tissue kit (Qiagen, Hilden, Germany), and the 16S rRNA gene was amplified using the primers 63f and 1387r (65). Amplicons were purified enzymatically with ExoSAP-It (Applied Biosystems, Waltham, MA) prior to Sanger sequencing (Eurofins Genomics, Louisville, KY) to confirm their species identity as P. aeruginosa. We did not detect pseudomonads other than P. aeruginosa. Furthermore, whole genomes were previously sequenced for all P. aeruginosa isolates collected from six study participants (patients 9, 24, 32, 33, 41, and 52 in Fig. S1) (10). We did not detect non-P. aeruginosa pseudomonads among whole-genome-sequenced isolates.

### FISH imaging of explanted obstructive sinus material.

When clinically indicated, obstructive sinus material was surgically removed from two study participants following sinonasal endoscopy and immediately fixed in 10% phosphate-buffered formalin (Fisher Scientific). Fixed samples were then rinsed and embedded for freezing in OCT compound (Tissue-Plus; Fisher HealthCare). Cryoprotected samples were sectioned at 10 μm on a Microm HM505E cryostat microtome (Microm International, Waldorf, Germany) and immobilized on poly-l-lysine-coated slides. In preparation for staining, slides were removed from the freezer and thawed at room temperature. Samples were permeabilized by incubating with lysozyme (10 mg/mL in 0.1 M Tris-HCl, 0.05 M EDTA) at 37°C for 3 h. The lysozyme solution was removed, and the samples were rinsed briefly with sterile RNase/DNase-free water. The samples were then dehydrated with increasing concentrations of ethanol (50%, 80%, and 100%) for 3 min per treatment as previously described (66) and then air dried at room temperature. FISH was performed using oligonucleotide probes directed toward 16S rRNA sequences specific to *Eubacteria* (Eub338; 5′-GCT GCC TCC CGT AGG AGT-3′) (67), S. aureus (Sau16S69; 5′-GAA GCA AGC TTC TCG TCC G-3′) (68), or P. aeruginosa (PsaerA; 5′-GGT AAC CGT CCC CCT TGC-3′) (66). Probes were synthesized by IDT (Coralville, IA) and 5′ labeled with the cyanine dye Cy3 (PsaerA and Sau16S69) or Cy5 (Eub338). Samples were incubated in hybridization buffer (0.9 M NaCl, 20 mM Tris-HCl [pH 7.6], 0.01% sodium dodecyl sulfate, 30% formamide) with the desired probe combinations for 1 h at 46°C. Samples were then washed with prewarmed washing buffer (20 mM Tris-HCl [pH 7.6], 0.01% sodium dodecyl sulfate, 112 mM NaCl) and incubated in washing buffer for 15 min at 48°C. Slides were then rinsed with sterile water, and the general DNA stain Hoechst trihydrochloride trihydrate was applied (1.0 μg/mL in phosphate-buffered saline [PBS]) for 10 min on ice. Slides were rinsed again with sterile water and left to air dry in a vertical position protected from light. When dry, samples were mounted with ProLong Gold antifade reagent (Life Technologies) for microscopy. Microscopy was performed on an Olympus FluoView FV1000 inverted confocal microscope using a 60× oil objective.

### Cytokine panels.

Cytokine levels in 54 sinus washes (stored frozen at −80°C) from 22 people were quantified using a Luminex MAGPIX system with either a Bio-Plex Pro human inflammation 24-Plex panel or a Bio-Plex Pro human Th17 cytokine panel 15-Plex panel (Bio-Rad, Hercules, CA, USA). Cytokines were omitted from further analyses if their concentration was close to the lower limit of detection in most samples, based on manufacturer-specified values and examination of the 5-parameter logistic (5pl) standard curves produced by the Bio-Plex Pro software. Seven cytokines were included in the predictor/responder analyses in Fig. 5. All cytokine concentrations were log transformed, except pentraxin 3 (PTX3), which was sufficiently normally distributed according to the Shapiro-Wilks test, without additional log transformation.

### Statistical analyses.

Statistical analyses were performed with GraphPad Prism version 9.1.2 or in RStudio version 1.1.456, and significance was determined at α = 0.05 unless otherwise specified. Relative abundances of taxa were transformed using the additive log ratio (ALR) transformation (69, 70). Alpha diversity values were calculated using the *diversity* function in the R package vegan v2.5-3.

The linear regression used to identify a relationship of FEV_1_ with CFRD status controlled for patient ID, age, and topical antibiotic usage. In Table 2, the linear regression used to identify a relationship of P. aeruginosa or other taxon relative abundance and CFRD status tested the top 15 taxa and controlled for patient ID, age, topical antibiotic usage, sinus or pulmonary exacerbation status, FEV_1_, and SNOT-22 and mLK scores.

Taxon co-occurrences in Fig. S2 were determined for the top 15 taxa by Pearson correlation across all samples (*n* = 101), and significance was determined after Holm-Bonferroni correction (*P* < 0.05). The Rs95 values included with taxon prevalence in Table S2 provide estimates that account for unequal sequencing depth using the binomial distribution. For example, in a sample with a read depth of 3,000, if the abundance of the taxon was 0.001, then according to the binomial distribution, the probability of not detecting this taxon (0 reads) is 0.0497 if the sample was resequenced to the same depth. Therefore, at least 1 read will be associated with this taxon in that sample, with a probability of 1 to 0.0497 = 0.9505, or >95% of the time.

The clustering analyses illustrated in Fig. 2A and B and Fig. S3 were performed with the “stats” package in R. The Manhattan distance between each sample was computed, and samples were hierarchically clustered based on the Ward’s minimum variance method. The taxa driving cluster formation were determined by calculating log(*R*^2^ ratios). Briefly, *R*^2^ (coefficient of determination) is a measure of separation between two groups of samples. The full *R*^2^ is defined as the *R*^2^ between two groups when all the taxa in the samples are included in the distance calculation. The reduced *R*^2^ is calculated when a specific taxon is excluded. When the ratio of reduced *R*^2^ to full *R*^2^ is less than 1, then that excluded taxon is considered a contributor to cluster separation.

In Fig. 2A, cluster multinomial log linear models were fit for covariates and clinical outcome variables to determine relationships between any of these variables with 16S microbiota profile cluster(s) using the R package “glm.” We used hierarchical cluster analysis with multinomial logistic regression (HCAMLR) as a method of associating predictors (covariates and clinical variables of interest) with the sets of clusters. In contrast to PERMANOVA, where a linear model is used to predict intersample distances, HCAMLR uses a multinomial logistic regression model to predict cluster membership. Hierarchical clusters were computed with Ward’s minimum variance algorithm using the Manhattan distance for computing intersample distances. The resultant tree was iteratively cut at increasing cluster counts *k*, from 2 to max_cuts=log_2_(num_samples). For each iteration with increasing *k*, a multinomial logistic regression model was fit for each of the *k* generated clusters to estimate the regression coefficients for each of the clinical predictors. At each iteration *k*, the most significant regression coefficient *P* value among the clusters *i* = 1 to *k* is retained for each of the predictors. After the last iteration (max_cuts), each of the predictor's “optimal cluster cutoffs” is independently identified by seeking the earliest iteration that produced the most significant *P* value for that predictor. The optimal cluster cutoff for a clinical predictor refers to the value of *k* that split the cohort into clusters with the most statistically significant heterogenous representation of the clinical predictors between clusters. The following covariates and clinical variables of interest were included in the logistic regression model to identify associations with cluster membership: isFemale, age_onenrollment, BMI_on_enrollment, CFRD, allergic_rhinitis, ever_on_topabx, ever_on_nasal_steroid, and sinus_exacerbation.

The PERMANOVA in Table S3 was performed with the *adonis2* function in vegan (*n* = 88 samples; 12,000 permutations) and specifying by = “margin” to assess the marginal effects of each term in the model. Age and CFRD status were inestimable in the PERMANOVA because they were confounded with patient ID.

Linear models in the predictor/responder analyses in Fig. 5 were calculated as previously described (22). Briefly, for both the sinus disease outcome and cytokine analyses, the following covariates were included: patient ID (to control for repeated measures within patients), age (age_onenrollment), sex (isFemale), BMI (BMI_on_enrollment), CFTR mutation (hmzg_mut508), CFRD diagnosis (CFRD), and current topical antibiotic use (current_topabx). To compute the associations, four pairs of linear regression models were fit. In panel A, diversity indices were tested as predictors of clinical variables with the model diversity indices = covariates + clinical variables. Conversely, diversity indices were tested as responding to clinical variables with the model clinical variables = covariates + diversity indices. In panel B, taxa are tested as predictors of clinical variables with taxa = covariates + clinical variables and as responders clinical variables = covariates + taxa. A similar analysis was performed with measures of cytokines in sinus wash. In panel C, diversity indices were tested as predictors of cytokine values with the model diversity indices = covariates + cytokines and as responders with cytokines = covariates + diversity indices. In panel D, taxa were tested as predictors with taxa = covariates + cytokines and as responders with cytokines = covariates + taxa. Taxa were represented as additive log ratio-transformed abundances. Cytokine concentrations were log transformed, except for pentraxin 3 (PTX3), which was sufficiently normally distributed. A *P* value of <0.025 in both the predictor and responder versions of the versions of the model (i.e., α = 0.05 for two simultaneous tests) was required for relationships to be summarized in Fig. 5. We inferred whether a variable was a predictor of or a responder to another variable by comparing *P* values from the pairs of models that used diversity or taxa as predictors of or responders to clinical variables or cytokines. We did not further investigate the biological basis of these predictor versus responder statistical associations experimentally.

### Data availability.

All V4 amplicon sequencing reads were deposited in NCBI’s SRA under BioProject no. PRJNA750353. Further information and requests for resources and reagents should be directed to and will be fulfilled by the lead contact, Jennifer Bomberger (jbomb@pitt.edu).
