# Supplementary material for: Low Diversity and Instability of the Sinus Microbiota over Time in Adults with Cystic Fibrosis
Source: Microbiol Spectr. 2022 Sep 12;10(5):e01251-22. doi: 10.1128/spectrum.01251-22 (PMC9603634; doi:10.1128/spectrum.01251-22)
Supplement: Supplemental file 1 — Supplemental material. Download spectrum.01251-22-s0001.pdf, PDF file, 0.5 MB [file spectrum.01251-22-s0001.pdf]

Supplemental figure legends

Figure S1. CF CRS microbial communities can be unstable, with many individuals switching between *Staphylococcus* spp. and *P. aeruginosa* at the greatest levels of relative abundance over time. Taxa bar plots depicting the percent of each taxon measured at clinic visit dates for each of the 27 study participants for whom we sequenced one microbiota sample (top) or longitudinal (bottom) samples. Colors representing each taxa can be matched by the legend on the right. *Staphylococcus* spp. is depicted in red, *P. aeruginosa* is represented by *Pseudomonadaceae* in peach and *Pseudomonas* spp. in dark teal. The X axis values (indicated beneath each stacked bar plot) depict the days since study enrollment. Black lines were drawn over the individual bar plots to indicate the degree of microbiota dissimilarity relative to the first time point. The units of dissimilarity were measured with the Manhattan distance and are annotated on the y-axis on the right. The taxa bar plot for Patient 9 was previously published in Armbruster et al., Cell Reports 2021 and is reproduced according to the CC BY-NC-ND 4.0 license.

Figure S2. Commensal taxa *Corynebacterium* spp. and *Dolosigranulum* spp. co-occur, whereas opportunistic pathogens *P. aeruginosa* and *Burkholderia* spp. are anticorrelated. Depicted in bold text and with a thick black box are the coefficients for relationships among the top 15 taxa that were statistically significant after controlling for multiple comparisons (Holm-Bonferroni adjusted p-value <0.05). In non-bold black text are individual associations that were statistically significant prior to correcting for multiple hypothesis testing ($p < 0.05$), but not after (Holm-Bonferroni adjusted p-value >0.05). Grey text indicates values that were not statistically significant.

Figure S3. Characterization of cluster assignment in Figure 2. A) The Calinski-Harabasz Pseudo-F statistic was calculated across all cluster cuts (k) to determine the optimal numbers of cluster cuts to use in Figure 2AB. Although at k = 5 cluster cuts, the clusters had the greatest inter-cluster separation, the cuts from k = 3 to 7 were not statistically significantly different from each other. Ultimately, the cluster cut at k = 3 was chosen because deeper cuts at k = 4 or k = 5 would have yielded cluster sizes too small to find statistically significant associations with the clinical or cytokine data. **B)** To determine which taxa influence the differentiation of clusters from one another (in Figure 2AB), taxa were iteratively evaluated for their contribution to pair-wise cluster separation by comparing the coefficient of determination (R^2) with (full) and without (reduced) the taxon of interest. The x-axis annotates this calculated metric: log(R^2 reduced / R^2 full). If excluding a taxon (reduced model) increases the separation between two clusters relative to its inclusion (full model), then it was an important clustering influencer, and the log ratio would

be <1. Log ratios greater than 1 indicate that the taxon added more noise (within cluster variance), thus reducing between cluster separation.

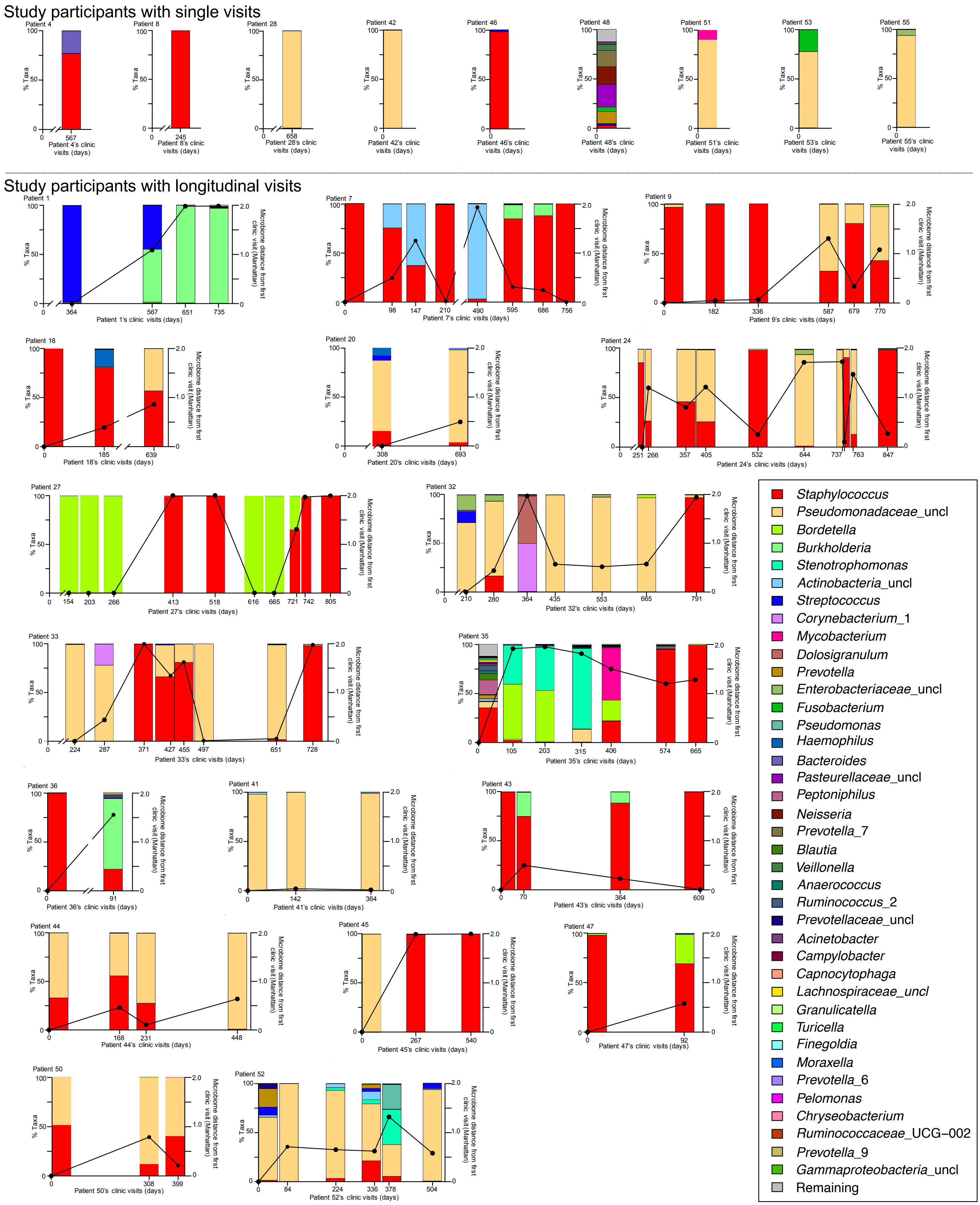
Study participants with single visits
Patient 4
Patient 4's clinic visits (days)
Patient 8
Patient 8's clinic visits (days)
Patient 28
Patient 28's clinic visits (days)
Patient 42
Patient 42's clinic visits (days)
Patient 46
Patient 46's clinic visits (days)
Patient 48
Patient 48's clinic visits (days)
Patient 51
Patient 51's clinic visits (days)
Patient 53
Patient 53's clinic visits (days)
Patient 55
Patient 55's clinic visits (days)
% Taxa
Study participants with longitudinal visits
Patient 1
Patient 1's clinic visits (days)
Patient 7
Patient 7's clinic visits (days)
Patient 9
Patient 9's clinic visits (days)
Patient 18
Patient 18's clinic visits (days)
Patient 20
Patient 20's clinic visits (days)
Patient 24
Patient 24's clinic visits (days)
Patient 27
Patient 27's clinic visits (days)
Patient 32
Patient 32's clinic visits (days)
Patient 33
Patient 33's clinic visits (days)
Patient 35
Patient 35's clinic visits (days)
Patient 36
Patient 36's clinic visits (days)
Patient 41
Patient 41's clinic visits (days)
Patient 43
Patient 43's clinic visits (days)
Patient 44
Patient 44's clinic visits (days)
Patient 45
Patient 45's clinic visits (days)
Patient 47
Patient 47's clinic visits (days)
Patient 50
Patient 50's clinic visits (days)
Patient 52
Patient 52's clinic visits (days)
Microbiome distance from first clinic visit (Manhattan)
Staphylococcus
Pseudomonadaceae_uncl
Bordetella
Burkholderia
Stenotrophomonas
Actinobacteria_uncl
Streptococcus
Corynebacterium_1
Mycobacterium
Dolosigranulum
Prevotella
Enterobacteriaceae_uncl
Fusobacterium
Pseudomonas
Haemophilus
Bacteroides
Pasteurellaceae_uncl
Peptoniphilus
Neisseria
Prevotella_7
Blautia
Veillonella
Anaerococcus
Ruminococcus_2
Prevotellaceae_uncl
Acinetobacter
Campylobacter
Capnocytophaga
Lachnospiraceae_uncl
Granulicatella
Turicella
Finegoldia
Moraxella
Prevotella_6
Pelomonas
Chryseobacterium
Ruminococcaceae_UCG−002
Prevotella_9
Gammaproteobacteria_uncl
Remaining

Figure S1

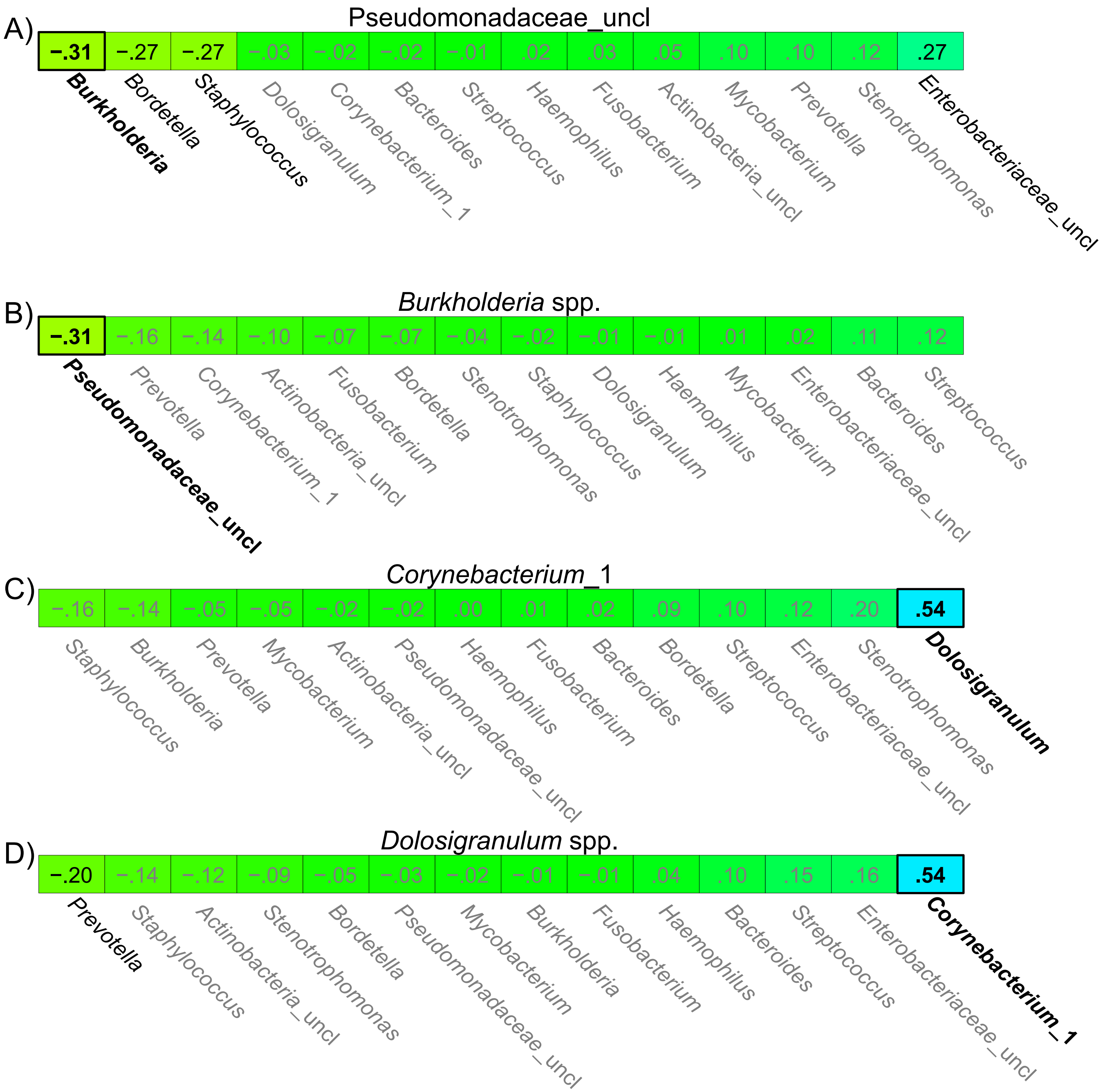
A)
Pseudomonadaceae_uncl
-.31 -.27 -.27 -.03 -.02 -.02 -.01 .02 .03 .05 .10 .10 .12 .27
Burkholderia
Bordetella
Staphylococcus
Dolosigranulum
Corynebacterium_1
Bacteroides
Streptococcus
Haemophilus
Fusobacterium
Actinobacteria_uncl
Mycobacterium
Prevotella
Stenotrophomonas
Enterobacteriaceae_uncl
B)
Burkholderia spp.
-.31 -.16 -.14 -.10 -.07 -.07 -.04 -.02 -.01 -.01 .01 .02 .11 .12
Pseudomonadaceae_uncl
Prevotella
Corynebacterium_1
Actinobacteria_uncl
Fusobacterium
Bordetella
Stenotrophomonas
Staphylococcus
Dolosigranulum
Haemophilus
Mycobacterium
Enterobacteriaceae_uncl
Bacteroides
Streptococcus
C)
Corynebacterium_1
-.16 -.14 -.05 -.05 -.02 -.02 .00 .01 .02 .09 .10 .12 .20 .54
Staphylococcus
Burkholderia
Prevotella
Mycobacterium
Actinobacteria_uncl
Pseudomonadaceae_uncl
Haemophilus
Fusobacterium
Bacteroides
Bordetella
Streptococcus
Enterobacteriaceae_uncl
Stenotrophomonas
Dolosigranulum
D)
Dolosigranulum spp.
-.20 -.14 -.12 -.09 -.05 -.03 -.02 -.01 -.01 .04 .10 .15 .16 .54
Prevotella
Staphylococcus
Actinobacteria_uncl
Stenotrophomonas
Bordetella
Pseudomonadaceae_uncl
Mycobacterium
Burkholderia
Fusobacterium
Haemophilus
Bacteroides
Streptococcus
Enterobacteriaceae_uncl
Corynebacterium_1

Figure S2

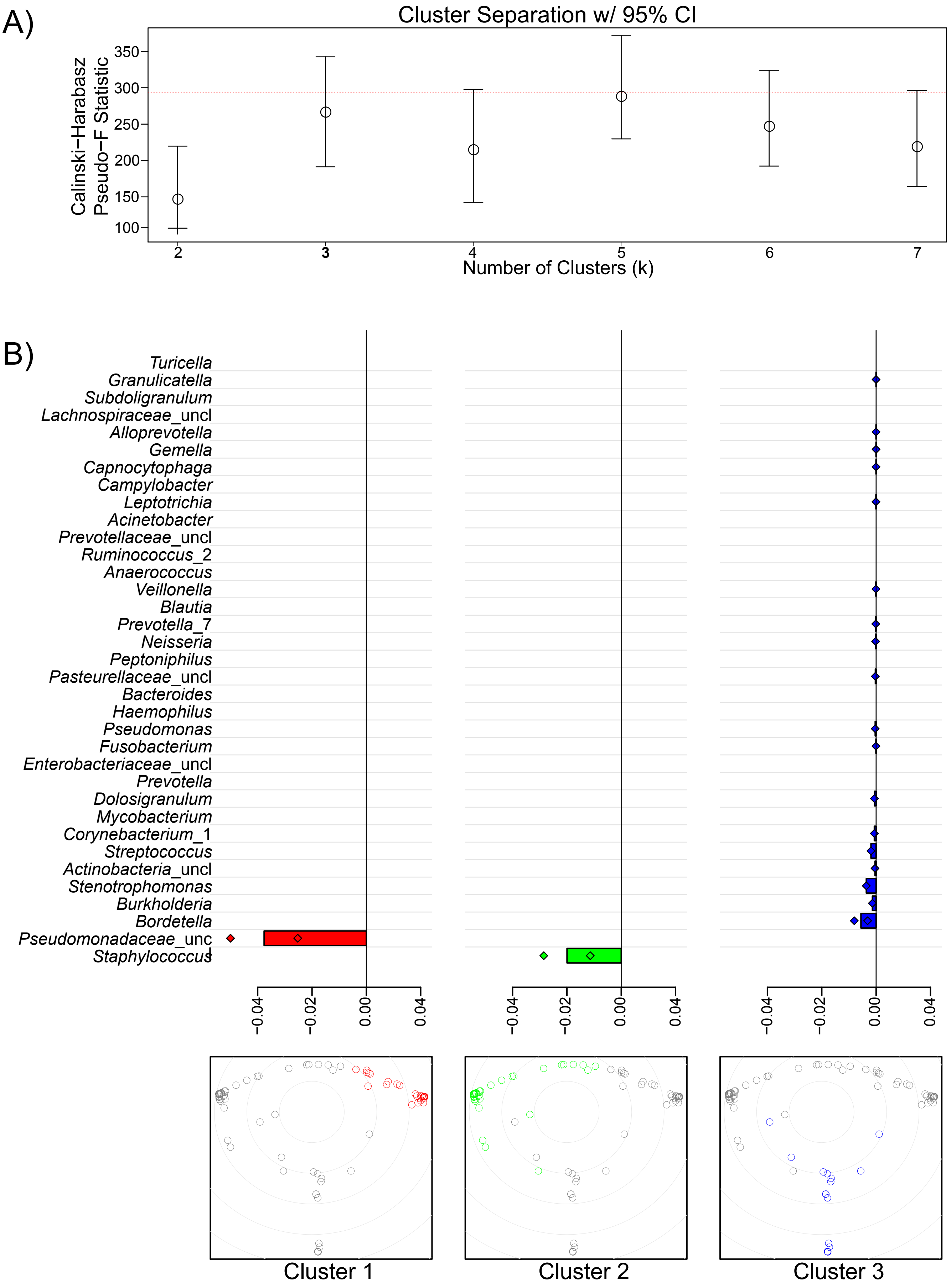
A)
Cluster Separation w/ 95% CI
Calinski−Harabasz Pseudo−F Statistic
350
300
250
200
150
100
2
3
4
5
6
7
Number of Clusters (k)
B)
Turicella
Granulicatella
Subdoligranulum
Lachnospiraceae_uncl
Alloprevotella
Gemella
Capnocytophaga
Campylobacter
Leptotrichia
Acinetobacter
Prevotellaceae_uncl
Ruminococcus_2
Anaerococcus
Veillonella
Blautia
Prevotella_7
Neisseria
Peptoniphilus
Pasteurellaceae_uncl
Bacteroides
Haemophilus
Pseudomonas
Fusobacterium
Enterobacteriaceae_uncl
Prevotella
Dolosigranulum
Mycobacterium
Corynebacterium_1
Streptococcus
Actinobacteria_uncl
Stenotrophomonas
Burkholderia
Bordetella
Pseudomonadaceae_unc
Staphylococcus
−0.04
−0.02
0.00
0.04
Cluster 1
Cluster 2
Cluster 3

Figure S3

Suplemental table 1. Diversity indices for each sequenced microbiota sample. The sample ID numbers listed correspond to the sample ID numbers in the metadata file (Supplemental table 4).

Sample ID	Shannon	Simpson	Evenness	Tail	Richness
0104.01.01.SINUS	0.10589483	0.03397046	0.05092465	0.287282	8
0104.01.02.SINUS	0.8179829	0.51611268	0.27780603	1.23346473	19
0104.01.03.SINUS	0.05154394	0.01166583	0.01750552	0.62712527	19
0104.01.04.SINUS	0.23749582	0.06885968	0.08769993	1.23729498	15
0104.04.01.SINUS	0.55157206	0.35447306	0.34271099	0.48696288	5
0104.07.01.SINUS	0.5631518	0.37064178	0.16249126	0.57228765	32
0104.07.02.SINUS	0.66782445	0.46875164	0.34319388	0.61981386	7
0104.07.03.SINUS	0.07910591	0.01762764	0.0211645	1.57350577	42
0104.07.04.SINUS	0.15674374	0.06603898	0.1426743	0.20127525	3
0104.07.05.SINUS	0.50714676	0.26517697	0.15957777	0.98347694	24
0104.07.06.SINUS	0.40024958	0.21522894	0.14779991	0.59603557	15
0104.07.07.SINUS	0.0121703	0.00264745	0.00679237	0.11119694	6
0104.07.08.SINUS	0.00778681	0.00148121	0.00324735	0.16889304	11
0104.08.01.SINUS	0.00432632	0.00088028	0.00312078	0.04532762	4
0104.09.01.SINUS	0.08402852	0.02486021	0.02470557	0.57216535	30
0104.09.02.SINUS	0.00142403	0.00028937	0.00205443	0.01202944	2
0104.09.03.SINUS	0.65588247	0.43865674	0.36605497	0.61119325	6
0104.09.04.SINUS	0.51529151	0.31923503	0.23451927	0.47379282	9
0104.09.05.SINUS	0.78229205	0.5148748	0.4366055	0.72857792	6
0104.09.06.SINUS	0.19234726	0.06978859	0.0605236	0.93817563	24
0104.18.01.SINUS	0.62057285	0.32374802	0.16939058	1.92072164	39
0104.18.02.SINUS	0.68887458	0.49111112	0.49691797	0.65939088	4
0104.18.03.SINUS	0.00836935	0.00174383	0.00467103	0.08647293	6
0104.20.01.SINUS	0.89683958	0.46001571	0.40816928	0.98816278	9
0104.20.04.SINUS	0.3885309	0.13060842	0.09652098	2.68572267	56
0104.24.01.SINUS	0.4094465	0.23690178	0.17782036	0.41456201	10
0104.24.02.SINUS	0.58119984	0.39109617	0.5290309	0.5167745	3
0104.24.03.SINUS	0.7110311	0.50035517	0.44178846	0.69425102	5
0104.24.04.SINUS	0.63897899	0.39483391	0.19850998	1.16587182	25
0104.24.05.SINUS	0.05833955	0.02057431	0.05310295	0.10332065	3
0104.24.07.SINUS	0.30522775	0.12649838	0.12283268	0.5061858	12
0104.24.08.SINUS	0.29419649	0.15405914	0.21221791	0.29673778	4
0104.24.09.SINUS	0.42659598	0.23561198	0.26505899	0.41326579	5
0104.24.10.SINUS	0.05857163	0.01761035	0.0363926	0.1517269	5
0104.24.12.SINUS	0.10335155	0.03279684	0.04703732	0.28326927	9
0104.27.01.SINUS	0.01002628	0.00181389	0.00289297	0.40264721	32
0104.27.02.SINUS	0.0079806	0.00161679	0.00410122	0.10310041	7
0104.27.03.SINUS	0.02148301	0.00472933	0.00932995	0.21063452	10
0104.27.04.SINUS	0.03243851	0.00824179	0.01305422	0.23774389	12
0104.27.05.SINUS	0.0210052	0.00565583	0.01515205	0.06893748	4
0104.27.06.SINUS	0.01964533	0.00571675	0.02834221	0.05354057	2
0104.27.07.SINUS	0.01097545	0.00263642	0.00791711	0.05137333	4
0104.27.08.SINUS	0.65581038	0.45334025	0.4074779	0.59864025	5
0104.27.09.SINUS	0.07297444	0.02729362	0.10527986	0.11763635	2
0104.27.10.SINUS	0.0177899	0.00440411	0.0128327	0.08401063	4
0104.28.01.SINUS	0.02275625	0.00551117	0.0116944	0.14182516	7
0104.32.01.SINUS	0.86733327	0.45454917	0.36170607	0.95381009	11
0104.32.02.SINUS	0.69004526	0.37816213	0.35461312	0.67418798	7

0104.32.03.SINUS	0.82344377	0.52160291	0.28489199	0.99783591	18
0104.32.04.SINUS	0.02379887	0.00605704	0.0122302	0.1255229	7
0104.32.05.SINUS	0.14457184	0.05562015	0.06278675	0.25443824	10
0104.32.06.SINUS	0.15334763	0.06380262	0.07880509	0.21475188	7
0104.32.07.SINUS	0.15684413	0.0605612	0.06811654	0.34175494	10
0104.33.01.SINUS	0.04310884	0.01254171	0.01491464	0.27159448	18
0104.33.02.SINUS	0.53595587	0.34303814	0.27542683	0.48392336	7
0104.33.03.SINUS	0.03000626	0.00746224	0.01365644	0.18039856	9
0104.33.04.SINUS	0.69416067	0.45336895	0.24500826	0.73161372	17
0104.33.05.SINUS	0.49254924	0.30718119	0.21391142	0.46863302	10
0104.33.06.SINUS	0.00719502	0.00139141	0.00280513	0.1381955	13
0104.33.07.SINUS	0.1788408	0.05278516	0.045953	1.98165623	49
0104.33.08.SINUS	0.08335648	0.02719195	0.03620126	0.25233094	10
0104.35.01.SINUS	0.89561934	0.52979487	0.32302639	1.00465728	16
0104.35.02.SINUS	0.85395521	0.52355481	0.26870382	1.25972202	24
0104.35.03.SINUS	0.75738899	0.31301841	0.19782187	3.56206679	46
0104.35.04.SINUS	1.24399943	0.62473957	0.31799389	3.13508178	50
0104.35.05.SINUS	0.3378025	0.10282788	0.08468384	2.96338754	54
0104.35.06.SINUS	2.63562916	0.83780344	0.62247588	13.0473956	69
0104.35.07.SINUS	0.06032969	0.01531783	0.02427845	0.38003598	12
0104.36.01.SINUS	0.03495463	0.0098714	0.01457721	0.17654247	11
0104.36.02.SINUS	0.83224584	0.42967049	0.42768976	1.14070365	7
0104.41.01.SINUS	0.10540975	0.04214031	0.07603706	0.15114284	4
0104.41.02.SINUS	0.01533826	0.0039999	0.01106422	0.05744511	4
0104.41.03.SINUS	0.06672001	0.02447487	0.09625663	0.11131475	2
0104.42.01.SINUS	0.01807113	0.00442783	0.01303556	0.09118583	4
0104.43.01.SINUS	0.02637757	0.00614694	0.00866394	0.3089862	21
0104.43.02.SINUS	0.59837818	0.37762027	0.21581931	0.67459121	16
0104.43.03.SINUS	0.36706226	0.20307205	0.14771672	0.42625751	12
0104.43.04.SINUS	0.03255379	0.0070786	0.01233538	0.45574127	14
0104.44.01.SINUS	0.64386079	0.44264048	0.21492601	0.659217	20
0104.44.02.SINUS	0.69408029	0.49392019	0.31588955	0.67550772	9
0104.44.03.SINUS	0.61347552	0.40354821	0.21224796	0.68952096	18
0104.44.05.SINUS	0.06140531	0.02132493	0.04429457	0.11321719	4
0104.45.01.SINUS	0.0081712	0.00171266	0.00456044	0.08352691	6
0104.45.02.SINUS	0.03262632	0.01001701	0.02027187	0.08118358	5
0104.45.05.SINUS	0.01537737	0.00400778	0.01399708	0.05487295	3
0104.46.01.SINUS	0.10567839	0.03882913	0.03902379	0.34435305	15
0104.47.01.SINUS	0.09856943	0.03622011	0.04280816	0.2160261	10
0104.47.02.SINUS	0.66867394	0.43215508	0.37319403	0.58890149	6
0104.48.01.SINUS	2.35363069	0.86435954	0.63379157	5.61677333	41
0104.50.01.SINUS	0.69408007	0.49985878	0.6317789	0.69914799	3
0104.50.02.SINUS	0.37886231	0.21033836	0.19469672	0.37760126	7
0104.50.03.SINUS	0.68887166	0.48359093	0.38446659	0.64854983	6
0104.51.01.SINUS	0.34527223	0.18211599	0.13461171	0.46116123	13
0104.52.01.SINUS	1.12138729	0.54057118	0.40445497	1.41079921	16
0104.52.02.SINUS	0.00520951	0.00105428	0.00290748	0.05420299	6
0104.52.03.SINUS	0.44870722	0.19058439	0.19487107	0.69406454	10
0104.52.05.SINUS	1.2623419	0.60501169	0.39720595	1.70145422	24
0104.52.06.SINUS	1.29989416	0.70018226	0.66801345	1.43817256	7
0104.52.07.SINUS	0.27742563	0.12400344	0.20012029	0.33000668	4
0104.53.01.SINUS	0.5450781	0.34868089	0.28011473	0.49035187	7
0104.55.01.SINUS	0.24599724	0.11865297	0.1774495	0.26771423	4

Supplemental table 2. Top 100 taxa identified in this study. The mean abundance per participant is averaged across all study participants, regardless of whether the taxon was detected. For participants with multiple study visits, the relative abundance of each taxon was first averaged across their visits. Number of participants is a count of the number of study participants with at least one study visit in which that taxon was identified. The prevalence is the percentage of patients with at least one study visit in which that taxon was identified. The Rs95 value is an estimate of mean abundance that takes into account the uneven sequencing depth of samples and is presented as a count of participants and prevalence based on this adjustment. All statistics for taxonnomic abundances are performed on additive log-transformed abundances.

Rank	Taxa	Mean abundance per participant	Number of participants	Prevalence (%)	Number of participants (Rs95)	Prevalence (Rs95;%)	Mean abundance across all samples	Standard Deviation	Standard Error
1	Staphylococcus	0.4118	25	92.6	23	85.2	0.43002067	0.424574076	0.0422467
2	Pseudomonadaceae_uncl	0.4077	25	92.6	21	77.8	0.348833988	0.41211472	0.041006947
3	Bordetella	0.043	15	55.6	10	37	0.069704213	0.230869672	0.022972391
4	Burkholderia	0.0275	8	29.6	4	14.8	0.038078581	0.163958354	0.016314466
5	Streptococcus	0.0146	19	70.4	13	48.1	0.01817556	0.107603503	0.010706949
6	Fusobacterium	0.01	6	22.2	2	7.4	0.002678309	0.022560873	0.002244891
7	Stenotrophomonas	0.0098	12	44.4	6	22.2	0.020840547	0.10566466	0.010514027
8	Actinobacteria_uncl	0.0093	13	48.1	8	29.6	0.020388974	0.116331921	0.011575459
9	Pasteurellaceae_uncl	0.0085	10	37	6	22.2	0.002338915	0.022733269	0.002262045
10	Bacteroides	0.0084	11	40.7	7	25.9	0.002373978	0.022598338	0.002248619
11	Mycobacterium	0.0069	3	11.1	2	7.4	0.006255698	0.053828728	0.005356159
12	Neisseria	0.0066	10	37	6	22.2	0.001873116	0.017635309	0.001754779
13	Prevotella_7	0.0062	6	22.2	4	14.8	0.00180565	0.016410278	0.001632884
14	Prevotella	0.0055	9	33.3	6	22.2	0.003978868	0.022749065	0.002263617
15	Haemophilus	0.0035	6	22.2	4	14.8	0.002634651	0.019099189	0.00190044
16	Enterobacteriaceae_uncl	0.0035	11	40.7	4	14.8	0.003576617	0.018490395	0.001839863
17	Corynebacterium_1	0.0033	15	55.6	9	33.3	0.007405359	0.053400466	0.005313545
18	Dolosigranulum	0.0024	4	14.8	2	7.4	0.004828915	0.048269142	0.004802959
19	Veillonella	0.0022	9	33.3	4	14.8	0.000647501	0.005971301	0.000594167
20	Leptotrichia	0.0017	5	18.5	2	7.4	0.000453501	0.004464456	0.00044423
21	Alloprevotella	0.0008	2	7.4	1	3.7	0.000202502	0.002025486	0.000201543
22	Gemella	0.0007	4	14.8	3	11.1	0.000202893	0.001922157	0.000191262
23	Lachnoanaerobaculum	0.0005	3	11.1	1	3.7	0.000147851	0.001463586	0.000145632
24	Peptoniphilus	0.0005	3	11.1	2	7.4	0.001928542	0.014726115	0.001465303
25	Selenomonas_3	0.0003	2	7.4	1	3.7	8.75489E-05	0.000857603	8.53347E-05
26	Capnocytophaga	0.0003	7	25.9	1	3.7	0.000212675	0.001449359	0.000144217
27	Prevotella_6	0.0003	4	14.8	2	7.4	0.00010341	0.000813092	8.09056E-05
28	Campylobacter	0.0003	4	14.8	3	11.1	0.000331682	0.001969071	0.00019593
29	Stomatobaculum	0.0003	3	11.1	1	3.7	7.58472E-05	0.000724581	7.20985E-05
30	Anaerococcus	0.0003	7	25.9	3	11.1	0.000557374	0.003150702	0.000313507
31	Prevotellaceae_uncl	0.0002	8	29.6	2	7.4	0.000517544	0.004888722	0.000486446
32	Pseudomonas	0.0002	12	44.4	5	18.5	0.002669558	0.02513086	0.002500614
33	Acinetobacter	0.0002	15	55.6	7	25.9	0.0004655	0.002066011	0.000205576
34	Moraxella	0.0002	10	37	6	22.2	0.000133847	0.000572013	5.69174E-05
35	Megasphaera	0.0002	4	14.8	1	3.7	4.51189E-05	0.000428852	4.26723E-05
36	Granulicatella	0.0001	6	22.2	1	3.7	0.000164862	0.001226152	0.000122007
37	Gammaproteobacteria_uncl	0.0001	8	29.6	4	14.8	7.13E-05	0.000275222	2.74E-05
38	Bergeyella	0.0001	3	11.1	1	3.7	2.43348E-05	0.000208357	2.07323E-05
39	Blautia	0.0001	8	29.6	3	11.1	0.000678114	0.00655322	0.00065207
40	Chryseobacterium	0.0001	6	22.2	4	14.8	8.801E-05	0.000541202	5.38517E-05
41	Sphingomonas	0.0001	11	40.7	7	25.9	5.25892E-05	0.00019488	1.93912E-05
42	Pelomonas	0.0001	10	37	5	18.5	0.000100349	0.000468823	4.66497E-05
43	Turicella	0.0001	6	22.2	2	7.4	0.000159799	0.001021605	0.000101654
44	Oribacterium	0.0001	2	7.4	2	7.4	1.91712E-05	0.000151526	1.50774E-05
45	Neisseriaceae_uncl	0.0001	5	18.5	2	7.4	4.27E-05	0.000268764	2.67E-05
46	Ruminococcus_2	0.0001	4	14.8	2	7.4	0.000556224	0.00546117	0.000543407
47	Lactobacillus	0.0001	7	25.9	4	14.8	5.14529E-05	0.000259294	2.58007E-05
48	Finegoldia	0.0001	5	18.5	2	7.4	0.000151581	0.000924509	9.19921E-05
49	Rothia	0	9	33.3	5	18.5	4.07843E-05	0.000131184	1.30533E-05
50	Aquabacterium	0	4	14.8	3	11.1	2.00704E-05	0.000150486	1.49739E-05
51	Bradyrhizobium	0	10	37	6	22.2	6.77824E-05	0.000255806	2.54537E-05
52	Micrococcus	0	8	29.6	2	7.4	5.24264E-05	0.000239931	2.3874E-05
53	[Eubacterium]_nodatum_grp	0	1	3.7	1	3.7	1.03E-05	0.000103496	1.03E-05
54	Bacilli_uncl	0	9	33.3	5	18.5	4.71E-05	0.000145319	1.45E-05
55	Comamonadaceae_uncl	0	5	18.5	4	14.8	7.56E-05	0.000504326	5.02E-05
56	Bacteria_uncl	0	8	29.6	4	14.8	4.18E-05	0.000146741	1.46E-05
57	Methylobacterium	0	9	33.3	6	22.2	4.05889E-05	0.000168225	1.6739E-05
58	Lachnospiraceae_uncl	0	7	25.9	2	7.4	0.000196502	0.00188629	0.000187693
59	Dialister	0	2	7.4	2	7.4	8.78323E-05	0.000525151	5.22544E-05
60	Alishewanella	0	4	14.8	1	3.7	1.1186E-05	8.23999E-05	8.1991E-06
61	Actinomyces	0	3	11.1	1	3.7	1.017E-05	6.53118E-05	6.49877E-06
62	unknown_uncl	0	3	11.1	1	3.7	3.06E-05	0.000176293	1.75E-05
63	Subdoligranulum	0	5	18.5	1	3.7	0.000167339	0.001415227	0.00014082
64	Porphyromonas	0	5	18.5	2	7.4	2.41443E-05	0.000159913	1.5912E-05
65	Hymenobacter	0	5	18.5	3	11.1	2.10241E-05	0.000105945	1.05419E-05

66	Alloiococcus	0	6	22.2	3	11.1	2.14639E-05	0.00012783	1.27196E-05
67	Domibacillus	0	1	3.7	1	3.7	3.03339E-05	0.000304852	3.03339E-05
68	Delftia	0	6	22.2	1	3.7	1.54631E-05	7.56312E-05	7.52559E-06
69	Ruminococcaceae_UCG_002	0	5	18.5	2	7.4	8.17E-05	0.000513844	5.11E-05
70	Prevotella_9	0	7	25.9	2	7.4	7.46306E-05	0.000568666	5.65844E-05
71	Prevotella_2	0	4	14.8	0	0	9.40291E-06	6.42449E-05	6.3926E-06
72	Janibacter	0	2	7.4	2	7.4	9.20962E-06	5.64448E-05	5.61646E-06
73	Alcaligenaceae_uncl	0	3	11.1	3	11.1	2.89E-05	0.000109037	1.08E-05
74	Pseudobutyrivibrio	0	6	22.2	3	11.1	0.000103679	0.000894836	8.90395E-05
75	Faecalibacterium	0	6	22.2	2	7.4	5.55362E-05	0.000342782	3.41081E-05
76	Sporobacter	0	1	3.7	1	3.7	5.71651E-06	5.74502E-05	5.71651E-06
77	Azomonas	0	7	25.9	2	7.4	1.67769E-05	5.25508E-05	5.229E-06
78	Anaerostipes	0	3	11.1	2	7.4	0.000132057	0.001290912	0.000128451
79	Micrococcaceae_uncl	0	6	22.2	1	3.7	1.69E-05	8.91E-05	8.87E-06
80	Fusicatenibacter	0	1	3.7	1	3.7	0.000138337	0.001390272	0.000138337
81	Sorangium	0	4	14.8	1	3.7	2.22701E-05	0.000139415	1.38723E-05
82	Bacillales_uncl	0	9	33.3	1	3.7	1.78E-05	8.07E-05	8.03E-06
83	Xanthomonadaceae_uncl	0	4	14.8	1	3.7	7.95E-06	4.95E-05	4.93E-06
84	Staphylococcaceae_uncl	0	7	25.9	1	3.7	1.39E-05	5.18E-05	5.15E-06
85	Massilia	0	3	11.1	1	3.7	1.43407E-05	7.2316E-05	7.19571E-06
86	Abiotrophia	0	5	18.5	1	3.7	9.92484E-06	3.74956E-05	3.73095E-06
87	Cloacibacterium	0	4	14.8	3	11.1	1.59258E-05	9.49309E-05	9.44598E-06
88	Ruminococcus_1	0	1	3.7	1	3.7	9.22517E-05	0.000894035	8.89598E-05
89	Actinobacillus	0	1	3.7	0	0	2.94234E-06	2.95702E-05	2.94234E-06
90	Facklamia	0	2	7.4	1	3.7	5.64365E-06	4.08389E-05	4.06363E-06
91	Devosia	0	2	7.4	1	3.7	5.64365E-06	4.08389E-05	4.06363E-06
92	Brevundimonas	0	3	11.1	1	3.7	1.62809E-05	0.00011187	1.11315E-05
93	Ruminococcaceae_uncl	0	2	7.4	1	3.7	2.00E-05	0.000140293	1.40E-05
94	Alphaproteobacteria_uncl	0	6	22.2	2	7.4	1.72E-05	9.01E-05	8.97E-06
95	Tepidiphilus	0	2	7.4	1	3.7	1.95942E-05	0.000186642	1.85716E-05
96	Conchiformibius	0	1	3.7	1	3.7	4.57391E-06	4.59672E-05	4.57391E-06
97	Candidate_division_SR1_uncl	0	2	7.4	0	0	3.12E-06	2.21E-05	2.20E-06
98	Sphingomonadales_uncl	0	6	22.2	0	0	3.76E-05	0.000236781	2.36E-05
99	Lachnoclostridium	0	8	29.6	3	11.1	3.29414E-05	0.00020429	2.03276E-05
100	Rheinheimera	0	3	11.1	0	0	7.04733E-06	4.60856E-05	4.58569E-06

Supplemental table 3. CF CRS microbial communities are highly individualized, but may share similarities during sinus exacerbation. PERMANOVA results describing the proportion of variance in sample composition attributable to variables tested ("source" of variation). One source contributed a significant amount of variance ($p < 0.05$; Patient ID). Whether or not a study participant was experiencing a sinus exacerbation had a non-statistically significant effect ($p < 0.1$). The remaining variables had non-significant effects ($p > 0.1$). The name of the variable as it appears in the metadata sheet is included in parentheses in the first column ("Source"). The model was run with 12000 permutations, using adonis2 with by = "margin". Significance levels were determined by the Pr(>F). ***: $p < 0.001$, **: $p < 0.01$, *: $p < 0.05$, º : $p < 0.1$, blank: $p > 0.1$.

Source	df	Sum of Squares (SS)	Mean Squares (MS)	F	R2	Pr(>F)	Significance
Patient ID (crs_ID)	26	54.159	2.08304	3.04244	0.51012	9.999e-05	***
Current topical antibiotic usage (current_topabx)	1	0.964	0.96358	1.40739	0.00908	0.22008	
Current sinus exacerbation (sinus_exacerbation)	1	1.724	1.72434	2.51854	0.01624	0.06599	º
Current pulmonary exacerbation (pulmonary_exacerbation)	1	0.163	0.16264	0.23754	0.00153	0.91471	
Residuals	67	45.872	0.68466		0.43207		
Total	96	106.169	1.10593		1		

Supplemental table 4. Metadata associated with each sequenced microbiota sample. See the codebook in Supplemental table 5 for a description of each variable.

SampleID	BarcodeSequence	LinkerPrimerSequence	participant_id	visit_number	days_since_first_visit	sex_is_female	age_onenrollment	BMI_on_enrollment	cfrd	transplant_prior_to_enrollment	hmzg_mut508	allergic_rhinitis	ever_on_topabx	ever_on_topvanco	ever_on_topgent
0104.01.01.SINUS	CGTTTGGAATGA	GTGTGYCAGCMGCCGCGGTAA	crs_01	1	0	0	23.94000053	24.90999985	0	0	0.5	0	1	1	1
0104.01.02.SINUS	AAGAACTCATGA	GTGTGYCAGCMGCCGCGGTAA	crs_01	2	77	0	23.94000053	24.90999985	0	0	0.5	0	1	1	1
0104.01.03.SINUS	TGATATCGTCTT	GTGTGYCAGCMGCCGCGGTAA	crs_01	3	161	0	23.94000053	24.90999985	0	0	0.5	0	1	1	1
0104.01.04.SINUS	CGGTGACCTACT	GTGTGYCAGCMGCCGCGGTAA	crs_01	4	245	0	23.94000053	24.90999985	0	0	0.5	0	1	1	1
0104.04.01.SINUS	AATTTAGGTAGG	GTGTGYCAGCMGCCGCGGTAA	crs_04	1	0	0	27.02000046	20.89999962	1	1	1	1	1	0	0
0104.07.08.SINUS	TTCGATGCCGCA	GTGTGYCAGCMGCCGCGGTAA	crs_07	1	0	1	43.56999969	24.84000015	1	0	1	1	1	1	1
0104.07.01.SINUS	AGAGGGTGATCG	GTGTGYCAGCMGCCGCGGTAA	crs_07	2	98	1	43.56999969	24.84000015	1	0	1	1	1	1	1
0104.07.02.SINUS	AGCTCTAGAAAC	GTGTGYCAGCMGCCGCGGTAA	crs_07	3	147	1	43.56999969	24.84000015	1	0	1	1	1	1	1
0104.07.03.SINUS	CTGACACGAATA	GTGTGYCAGCMGCCGCGGTAA	crs_07	4	210	1	43.56999969	24.84000015	1	0	1	1	1	1	1
0104.07.04.SINUS	GCTGCCCACCTA	GTGTGYCAGCMGCCGCGGTAA	crs_07	5	490	1	43.56999969	24.84000015	1	0	1	1	1	1	1
0104.07.05.SINUS	GCGTTTGCTAGC	GTGTGYCAGCMGCCGCGGTAA	crs_07	6	595	1	43.56999969	24.84000015	1	0	1	1	1	1	1
0104.07.06.SINUS	AGATCGTGCCTA	GTGTGYCAGCMGCCGCGGTAA	crs_07	7	686	1	43.56999969	24.84000015	1	0	1	1	1	1	1
0104.07.07.SINUS	AGATCGTGCCTA	GTGTGYCAGCMGCCGCGGTAA	crs_07	8	756	1	43.56999969	24.84000015	1	0	1	1	1	1	1
0104.08.01.SINUS	AATGGTTCAGCA	GTGTGYCAGCMGCCGCGGTAA	crs_08	1	0	1	24.20999908	19.52000046	0	0	0.5	1	1	0	1
0104.09.06.SINUS	GAACCAGTACTC	GTGTGYCAGCMGCCGCGGTAA	crs_09	1	0	1	25.79000092	28.76000023	1	NA	0.5	0	1	0	0
0104.09.01.SINUS	CGCACCCATACA	GTGTGYCAGCMGCCGCGGTAA	crs_09	2	182	1	25.79000092	28.76000023	1	NA	0.5	0	1	0	0
0104.09.02.SINUS	GTGCCATAATCG	GTGTGYCAGCMGCCGCGGTAA	crs_09	3	336	1	25.79000092	28.76000023	1	NA	0.5	0	1	0	0
0104.09.03.SINUS	CTACAGGGTCTC	GTGTGYCAGCMGCCGCGGTAA	crs_09	4	587	1	25.79000092	28.76000023	1	NA	0.5	0	1	0	0
0104.09.04.SINUS	CTACAGGGTCTC	GTGTGYCAGCMGCCGCGGTAA	crs_09	5	679	1	25.79000092	28.76000023	1	NA	0.5	0	1	0	0
0104.09.05.SINUS	CTTGGAGGCTTA	GTGTGYCAGCMGCCGCGGTAA	crs_09	6	770	1	25.79000092	28.76000023	1	NA	0.5	0	1	0	0
0104.18.03.SINUS	CACGTTTATTCC	GTGTGYCAGCMGCCGCGGTAA	crs_18	1	0	1	27.68000031	19.87999916	1	0	1	0	1	0	0
0104.18.01.SINUS	TAATCGGTGCCA	GTGTGYCAGCMGCCGCGGTAA	crs_18	2	185	1	27.68000031	19.87999916	1	0	1	0	1	0	0
0104.18.02.SINUS	CGGGACACCCGA	GTGTGYCAGCMGCCGCGGTAA	crs_18	3	639	1	27.68000031	19.87999916	1	0	1	0	1	0	0
0104.20.01.SINUS	GAAGAGGGTTGA	GTGTGYCAGCMGCCGCGGTAA	crs_20	1	0	1	23.06999969	19.54000092	0	0	1	0	1	0	1
0104.20.04.SINUS	TTACACAAAGGC	GTGTGYCAGCMGCCGCGGTAA	crs_20	2	385	1	23.06999969	19.54000092	0	0	1	0	1	0	1
0104.24.01.SINUS	GTTAACTTACTA	GTGTGYCAGCMGCCGCGGTAA	crs_24	1	0	1	27.62000084	24.13999939	1	0	1	1	1	1	1
0104.24.02.SINUS	GTTAACTTACTA	GTGTGYCAGCMGCCGCGGTAA	crs_24	2	15	1	27.62000084	24.13999939	1	0	1	1	1	1	1
0104.24.03.SINUS	CGTATAAATGCG	GTGTGYCAGCMGCCGCGGTAA	crs_24	3	106	1	27.62000084	24.13999939	1	0	1	1	1	1	1
0104.24.04.SINUS	ATGCTGCAACAC	GTGTGYCAGCMGCCGCGGTAA	crs_24	4	154	1	27.62000084	24.13999939	1	0	1	1	1	1	1
0104.24.05.SINUS	ACTCGCTCGCTG	GTGTGYCAGCMGCCGCGGTAA	crs_24	5	281	1	27.62000084	24.13999939	1	0	1	1	1	1	1
0104.24.07.SINUS	CGTCCGTATGAA	GTGTGYCAGCMGCCGCGGTAA	crs_24	6	393	1	27.62000084	24.13999939	1	0	1	1	1	1	1
0104.24.10.SINUS	ACGTGAGGAACG	GTGTGYCAGCMGCCGCGGTAA	crs_24	7	486	1	27.62000084	24.13999939	1	0	1	1	1	1	1
0104.24.08.SINUS	GTTGTTCTGGGA	GTGTGYCAGCMGCCGCGGTAA	crs_24	8	491	1	27.62000084	24.13999939	1	0	1	1	1	1	1
0104.24.09.SINUS	GTTGTTCTGGGA	GTGTGYCAGCMGCCGCGGTAA	crs_24	9	512	1	27.62000084	24.13999939	1	0	1	1	1	1	1
0104.24.12.SINUS	CATATAGCCCGA	GTGTGYCAGCMGCCGCGGTAA	crs_24	10	596	1	27.62000084	24.13999939	1	0	1	1	1	1	1
0104.27.01.SINUS	ACGCTTAACGAC	GTGTGYCAGCMGCCGCGGTAA	crs_27	1	0	1	23.45000076	19.15999985	1	0	0.5	0	1	0	1
0104.27.02.SINUS	TACGGATTATGG	GTGTGYCAGCMGCCGCGGTAA	crs_27	2	49	1	23.45000076	19.15999985	1	0	0.5	0	1	0	1
0104.27.03.SINUS	ATACATGCAAGA	GTGTGYCAGCMGCCGCGGTAA	crs_27	3	112	1	23.45000076	19.15999985	1	0	0.5	0	1	0	1
0104.27.04.SINUS	CTTAGTGCAGAA	GTGTGYCAGCMGCCGCGGTAA	crs_27	4	259	1	23.45000076	19.15999985	1	0	0.5	0	1	0	1
0104.27.05.SINUS	AATCTTGCGCCG	GTGTGYCAGCMGCCGCGGTAA	crs_27	5	364	1	23.45000076	19.15999985	1	0	0.5	0	1	0	1
0104.27.06.SINUS	AGGATCAGGGAA	GTGTGYCAGCMGCCGCGGTAA	crs_27	6	462	1	23.45000076	19.15999985	1	0	0.5	0	1	0	1
0104.27.07.SINUS	AATAACTAGGGT	GTGTGYCAGCMGCCGCGGTAA	crs_27	7	511	1	23.45000076	19.15999985	1	0	0.5	0	1	0	1
0104.27.08.SINUS	AATAACTAGGGT	GTGTGYCAGCMGCCGCGGTAA	crs_27	8	567	1	23.45000076	19.15999985	1	0	0.5	0	1	0	1
0104.27.09.SINUS	TATTGCAGCAGC	GTGTGYCAGCMGCCGCGGTAA	crs_27	9	588	1	23.45000076	19.15999985	1	0	0.5	0	1	0	1
0104.27.10.SINUS	TGATGTGCTAAG	GTGTGYCAGCMGCCGCGGTAA	crs_27	10	651	1	23.45000076	19.15999985	1	0	0.5	0	1	0	1
0104.28.01.SINUS	CTTATTAAACGT	GTGTGYCAGCMGCCGCGGTAA	crs_28	1	0	0	28.36000061	20.34000015	0	0	0	1	1	0	0
0104.32.01.SINUS	TATTTGATTGGT	GTGTGYCAGCMGCCGCGGTAA	crs_32	1	0	0	26.54000092	18.45000076	1	0	0.5	1	1	0	1
0104.32.02.SINUS	TGTCAAAGTGAC	GTGTGYCAGCMGCCGCGGTAA	crs_32	2	70	0	26.54000092	18.45000076	1	0	0.5	1	1	0	1
0104.32.03.SINUS	CTATGTATTAGT	GTGTGYCAGCMGCCGCGGTAA	crs_32	3	154	0	26.54000092	18.45000076	1	0	0.5	1	1	0	1
0104.32.04.SINUS	CTATGTATTAGT	GTGTGYCAGCMGCCGCGGTAA	crs_32	4	225	0	26.54000092	18.45000076	1	0	0.5	1	1	0	1
0104.32.05.SINUS	ACTCCCGTGTGA	GTGTGYCAGCMGCCGCGGTAA	crs_32	5	343	0	26.54000092	18.45000076	1	0	0.5	1	1	0	1
0104.32.06.SINUS	CGGTATAGCAAT	GTGTGYCAGCMGCCGCGGTAA	crs_32	6	455	0	26.54000092	18.45000076	1	0	0.5	1	1	0	1
0104.32.07.SINUS	CGGTATAGCAAT	GTGTGYCAGCMGCCGCGGTAA	crs_32	7	581	0	26.54000092	18.45000076	1	0	0.5	1	1	0	1
0104.33.01.SINUS	ACTTGGTGTAAG	GTGTGYCAGCMGCCGCGGTAA	crs_33	1	0	1	27.03000069	21.56999969	0	0	1	0	1	0	0
0104.33.02.SINUS	ACTTGGTGTAAG	GTGTGYCAGCMGCCGCGGTAA	crs_33	2	63	1	27.03000069	21.56999969	0	0	1	0	1	0	0
0104.33.03.SINUS	ATTACGTATCAT	GTGTGYCAGCMGCCGCGGTAA	crs_33	3	147	1	27.03000069	21.56999969	0	0	1	0	1	0	0
0104.33.04.SINUS	ATAGGAATAACC	GTGTGYCAGCMGCCGCGGTAA	crs_33	4	203	1	27.03000069	21.56999969	0	0	1	0	1	0	0
0104.33.05.SINUS	ATAGGAATAACC	GTGTGYCAGCMGCCGCGGTAA	crs_33	5	231	1	27.03000069	21.56999969	0	0	1	0	1	0	0
0104.33.06.SINUS	ATAGGAATAACC	GTGTGYCAGCMGCCGCGGTAA	crs_33	6	273	1	27.03000069	21.56999969	0	0	1	0	1	0	0
0104.33.07.SINUS	TGTGCACGCCAT	GTGTGYCAGCMGCCGCGGTAA	crs_33	7	427	1	27.03000069	21.56999969	0	0	1	0	1	0	0
0104.33.08.SINUS	TGTGCACGCCAT	GTGTGYCAGCMGCCGCGGTAA	crs_33	8	504	1	27.03000069	21.56999969	0	0	1	0	1	0	0
0104.35.06.SINUS	TATCCAAGCGCA	GTGTGYCAGCMGCCGCGGTAA	crs_35	1	0	1	35.02999878	25.04999924	0	0	1	1	1	0	1
0104.35.01.SINUS	AGAGCCAAGAGC	GTGTGYCAGCMGCCGCGGTAA	crs_35	2	105	1	35.02999878	25.04999924	0	0	1	1	1	0	1
0104.35.02.SINUS	GGTGAGCAAGCA	GTGTGYCAGCMGCCGCGGTAA	crs_35	3	203	1	35.02999878	25.04999924	0	0	1	1	1	0	1
0104.35.03.SINUS	TTGCGGACCCTA	GTGTGYCAGCMGCCGCGGTAA	crs_35	5	315	1	35.02999878	25.04999924	0	0	1	1	1	0	1
0104.35.04.SINUS	TTGCGGACCCTA	GTGTGYCAGCMGCCGCGGTAA	crs_35	6	406	1	35.02999878	25.04999924	0	0	1	1	1	0	1
0104.35.05.SINUS	GTCGTCCAAATG	GTGTGYCAGCMGCCGCGGTAA	crs_35	7	574	1	35.02999878	25.04999924	0	0	1	1	1	0	1
0104.35.07.SINUS	TGCACAGTCGCT	GTGTGYCAGCMGCCGCGGTAA	crs_35	8	665	1	35.02999878	25.04999924	0	0	1	1	1	0	1
0104.36.01.SINUS	GCTGCGTATACC	GTGTGYCAGCMGCCGCGGTAA	crs_36	1	0	0	23.30999947	26.72999954	0	0	0.5	0	1	0	0
0104.36.02.SINUS	GCTGCGTATACC	GTGTGYCAGCMGCCGCGGTAA	crs_36	2	91	0	23.30999947	26.72999954	0	0	0.5	0	1	0	0
0104.41.01.SINUS	TACTAACGCGGT	GTGTGYCAGCMGCCGCGGTAA	crs_41	1	0	1	27.56999969	18.35000038	1	0	1	0	1	0	1
0104.41.02.SINUS	GCGATCACACCT	GTGTGYCAGCMGCCGCGGTAA	crs_41	2	142	1	27.56999969	18.35000038	1	0	1	0	1	0	1
0104.41.03.SINUS	CAAACGCACTAA	GTGTGYCAGCMGCCGCGGTAA	crs_41	3	364	1	27.56999969	18.35000038	1	0	1	0	1	0	1
0104.42.01.SINUS	GTCATGCTCCAG	GTGTGYCAGCMGCCGCGGTAA	crs_42	1	0	1	38.58000183	30.06999969	1	1	NA	0	1	0	0
0104.43.01.SINUS	GAGATACAGTTC	GTGTGYCAGCMGCCGCGGTAA	crs_43	1	0	0	33.22999954	22.01000023	0	0	0	1	0	0	0
0104.43.02.SINUS	GTGGAGTCTCAT	GTGTGYCAGCMGCCGCGGTAA	crs_43	2	70	0	33.22999954	22.01000023	0	0	0	1	0	0	0
0104.43.03.SINUS	ACCTTACACCTT	GTGTGYCAGCMGCCGCGGTAA	crs_43	3	364	0	33.22999954	22.01000023	0	0	0	1	0	0	0
0104.43.04.SINUS	TAATCTCGCCGG	GTGTGYCAGCMGCCGCGGTAA	crs_43	4	609	0	33.22999954	22.01000023	0	0	0	1	0	0	0
0104.44.01.SINUS	GGAGAACGACAC	GTGTGYCAGCMGCCGCGGTAA	crs_44	1	0	1	26.19000053	19.65999985	0	NA	1	1	1	0	0
0104.44.02.SINUS	GTACTCGAACCA	GTGTGYCAGCMGCCGCGGTAA	crs_44	2	168	1	26.19000053	19.65999985	0	NA	1	1	1	0	0
0104.44.03.SINUS	GTACTCGAACCA	GTGTGYCAGCMGCCGCGGTAA	crs_44	3	231	1	26.19000053	19.65999985	0	NA	1	1	1	0	0
0104.44.05.SINUS	TCCGGCGGGCAA	GTGTGYCAGCMGCCGCGGTAA	crs_44	4	448	1	26.19000053	19.65999985	0	NA	1	1	1	0	0
0104.45.01.SINUS	TCCATACCGGAA	GTGTGYCAGCMGCCGCGGTAA	crs_45	1	0	0	24.20999908	19.59000015	0	0	0.5	1	1	0	1
0104.45.02.SINUS	TCCATACCGGAA	GTGTGYCAGCMGCCGCGGTAA	crs_45	2	267	0	24.20999908	19.59000015	0	0	0.5	1	1	0	1
0104.45.05.SINUS	CCTAACGGTCCA	GTGTGYCAGCMGCCGCGGTAA	crs_45	3	540	0	24.20999908	19.59000015	0	0	0.5	1	1	0	1
0104.46.01.SINUS	GTTCATTAAACT	GTGTGYCAGCMGCCGCGGTAA	crs_46	1	0	1	36.54000092	24	1	0	0.5	0	0	0	0
0104.47.01.SINUS	CTCGCCCTCGCC	GTGTGYCAGCMGCCGCGGTAA	crs_47	1	0	1	26.18000031	17.88999939	0	0	1	1	1	0	0
0104.47.02.SINUS	TCTCTTTCGACA	GTGTGYCAGCMGCCGCGGTAA	crs_47	2	92	1	26.18000031	17.88999939	0	0	1	1	1	0	0
0104.48.01.SINUS	CTTGATTCTTGA	GTGTGYCAGCMGCCGCGGTAA	crs_48	1	0	0	31.75	26.5	0	0	1	0	1	0	0
0104.50.01.SINUS	GTGCCGGCCGAC	GTGTGYCAGCMGCCGCGGTAA	crs_50	1	0	1	30.71999931	22.11000061	0	0	0.5	1	0	0	0

0104.50.02.SINUS	CCTTGACCGATG	GTGTGYCAGCMGCCGCGGTAA	crs_50	2	308	1	30.71999931	22.11000061	0	0	0.5	1	0	0	0
0104.50.03.SINUS	CAAACTGCGTTG	GTGTGYCAGCMGCCGCGGTAA	crs_50	3	399	1	30.71999931	22.11000061	0	0	0.5	1	0	0	0
0104.51.01.SINUS	GTTGATACGATG	GTGTGYCAGCMGCCGCGGTAA	crs_51	1	0	1	36.16999817	18.60000038	0	0	1	1	0	0	0
0104.52.01.SINUS	CTATATTATCCG	GTGTGYCAGCMGCCGCGGTAA	crs_52	1	0	1	35.83000183	23.03000069	1	0	1	0	1	0	1
0104.52.02.SINUS	ACCGAACAATCC	GTGTGYCAGCMGCCGCGGTAA	crs_52	2	84	1	35.83000183	23.03000069	1	0	1	0	1	0	1
0104.52.03.SINUS	ACGGTACCCTAC	GTGTGYCAGCMGCCGCGGTAA	crs_52	3	224	1	35.83000183	23.03000069	1	0	1	0	1	0	1
0104.52.05.SINUS	ACCTACTTGTCT	GTGTGYCAGCMGCCGCGGTAA	crs_52	4	336	1	35.83000183	23.03000069	1	0	1	0	1	0	1
0104.52.06.SINUS	ACTGTGACGTCC	GTGTGYCAGCMGCCGCGGTAA	crs_52	5	378	1	35.83000183	23.03000069	1	0	1	0	1	0	1
0104.52.07.SINUS	CATGTCTTCCAT	GTGTGYCAGCMGCCGCGGTAA	crs_52	6	504	1	35.83000183	23.03000069	1	0	1	0	1	0	1
0104.53.01.SINUS	GTAGTAGACCAT	GTGTGYCAGCMGCCGCGGTAA	crs_53	1	0	1	19.67000008	20.77000046	0	0	0.5	1	0	0	0
0104.55.01.SINUS	CCTCCGTCATGG	GTGTGYCAGCMGCCGCGGTAA	crs_55	1	0	0	40	22.20000076	1	0	0.5	1	1	0	1

ever_on_top_mupirocin	ever_on_top_cipro	ever_on_nasal_steroid	ever_on_chronic_oral_steroid	sinus_exacerbation	pulmonary_exacerbation	on_nasal_cannula_oxygen	interim_hos	hospital_days	transplant_at_visit	visit_bmi	samp_loc	ppFEV1	ppFEF25-75	snot22	mlk	sputum_pa
0	0	1	0	0	1	0	0	NA	0	25.40999985	Maxillary	94	NA	20	6	NA
0	0	1	0	0	NA	NA	NA	NA	0	NA	Maxillary	94	74	5	4	0
0	0	1	0	0	NA	NA	NA	NA	0	NA	Ethmoid	101	81	18	0	0
0	0	1	0	0	NA	NA	NA	NA	NA	NA	Ethmoid	109	91	9	0	0
1	1	0	1	0	0	0	0	NA	1	20.04999924	Maxillary	77	90	61	12	NA
0	0	0	1	0	1	0	NA	NA	0	25.04999924	Ethmoid	66	27	62	10	0
0	0	0	1	0	1	0	1	9	0	23.43000031	Ethmoid	70	31	48	12	0
0	0	0	1	0	1	0	0	NA	0	22.80999947	Ethmoid	65	34	34	8	0
0	0	0	1	0	0	0	0	NA	0	22.79000092	Ethmoid	63	26	59	8	0
0	0	0	1	0	0	0	0	NA	0	24.70999908	Ethmoid	63	29	54	6	0
0	0	0	1	1	0	0	0	NA	0	25.95000076	Ethmoid	63	24	54	10	0
0	0	0	1	1	1	0	0	NA	0	25.37000084	Ethmoid	66	28	58	10	0
0	0	0	1	1	0	1	0	NA	0	26.40999985	Ethmoid	56	21	55	10	0
1	0	0	0	0	1	0	1	3	0	18.10000038	Maxillary	76	62	47	12	0
1	1	0	0	0	0	0	0	NA	0	28.76000023	Ethmoid	100	79	67	16	1
1	1	0	0	0	0	0	1	10	0	29.70000076	Ethmoid	92	63	83	16	1
1	1	0	0	0	0	0	1	13	0	30.86000061	Ethmoid	104	70	85	16	1
1	1	0	0	1	1	0	0	NA	0	33.09000015	Ethmoid	83	46	83	8	NA
1	1	0	0	1	0	0	1	3	0	33.54999924	Ethmoid	101	72	81	8	1
1	1	0	0	1	0	0	0	NA	0	33.91999817	Ethmoid	98	79	96	NA	1
1	1	0	1	0	0	0	0	NA	0	19.87999916	Ethmoid	54	19	30	14	0
1	1	0	1	0	0	0	1	13	0	22.06999969	Maxillary	51	18	18	6	NA
1	1	0	1	1	0	0	1	23	0	19.87999916	Ethmoid	47	15	63	8	1
1	1	1	0	1	0	0	0	NA	0	19.36000061	Maxillary	71	64	48	8	1
1	1	1	0	0	1	0	0	NA	0	19.03000069	Maxillary	66	85	51	4	0
0	0	0	0	1	1	1	0	NA	0	NA	Ethmoid	27	8	43	12	0
0	0	0	0	0	1	1	0	NA	0	22.52000046	Ethmoid	27	8	39	8	1
0	0	0	0	0	0	1	0	NA	0	23.10000038	Ethmoid	34	10	50	10	1
0	0	0	0	1	1	1	1	4	0	21.94000053	Ethmoid	31	11	49	12	0
0	0	0	0	1	1	1	1	3	0	21.57999992	Maxillary	25	8	40	12	0
0	0	0	0	1	1	1	0	NA	0	24.34000015	Ethmoid	30	11	43	10	1
0	0	0	0	0	1	1	0	NA	0	23.04000092	Ethmoid	27	10	33	10	1
0	0	0	0	1	1	1	0	NA	0	23.04000092	Ethmoid	25	8	45	10	1
0	0	0	0	0	1	1	0	NA	0	24.34000015	Maxillary	28	10	26	10	1
0	0	0	0	1	1	1	0	NA	0	23.97999954	Ethmoid	21	7	45	12	1
1	1	0	1	0	1	1	1	NA	0	18.47999954	Ethmoid	38	42	12	8	NA
1	1	0	1	0	0	1	0	NA	0	17.85000038	Ethmoid	26	25	30	12	0
1	1	0	1	0	1	1	0	NA	0	18.81999969	Ethmoid	27	16	14	8	0
1	1	0	1	1	1	1	0	NA	0	17.85000038	Ethmoid	28	18	29	12	0
1	1	0	1	0	0	1	1	6	0	19.69000053	Maxillary	37	35	21	10	0
1	1	0	1	0	1	1	1	4	0	18.81999969	Ethmoid	27	24	26	6	0
1	1	0	1	0	0	1	0	NA	0	NA	Ethmoid	32	28	15	6	0
1	1	0	1	0	1	1	0	NA	0	18.81999969	Ethmoid	25	40	18	6	0
1	1	0	1	1	1	1	1	3	0	19.84000015	Ethmoid	22	19	18	8	0
1	1	0	1	0	1	1	0	NA	0	19.69000053	Ethmoid	33	10	8	6	0
0	1	1	1	0	1	0	1	3	0	22.95000076	Maxillary	42	14	21	6	NA
1	1	1	0	1	0	0	0	NA	0	19.68000031	Maxillary	60	32	60	8	1
1	1	1	0	0	1	0	1	1	0	20.55999947	Maxillary	67	34	36	8	1
1	1	1	0	0	0	0	0	NA	0	19.95999908	Maxillary	61	30	59	4	1
1	1	1	0	1	1	0	0	NA	0	19.19000053	Maxillary	77	18	52	NA	1
1	1	1	0	0	0	0	1	5	0	19.44000053	Maxillary	NA	NA	62	8	NA
1	1	1	0	1	0	0	1	10	0	17.31999969	Maxillary	58	25	52	10	1
1	1	1	0	1	1	0	0	NA	0	17.95000076	Ethmoid	57	20	74	8	1
1	1	1	1	1	0	0	0	NA	0	22.05999947	Maxillary	88	59	0	10	1
1	1	1	1	0	0	0	0	NA	0	21.71999931	Maxillary	91	71	0	6	1
1	1	1	1	1	1	0	1	1	0	21.94000053	Maxillary	92	66	0	10	1
1	1	1	1	1	0	0	0	NA	0	22.59000015	Maxillary	83	59	0	4	NA
1	1	1	1	0	1	0	0	NA	0	22.64999962	Ethmoid	81	54	3	4	1
1	1	1	1	0	0	0	0	NA	0	22.25	Ethmoid	83	55	0	3	1
1	1	1	1	0	0	0	0	NA	0	NA	Ethmoid	82	52	1	2	1
1	1	1	1	0	0	0	0	NA	0	23.11000061	Ethmoid	94	58	1	4	1
0	0	1	0	0	0	0	0	NA	0	25.04999924	Maxillary	94	55	29	6	0
0	0	1	0	0	0	0	0	NA	0	23.87999916	Maxillary	92	55	16	6	0
0	0	1	0	0	0	0	0	NA	0	24.20999908	Maxillary	91	58	25	6	NA
0	0	1	0	1	1	0	0	NA	0	24.65999985	Ethmoid	86	49	48	8	0
0	0	1	0	1	0	0	0	NA	0	24.80999947	Ethmoid	94	59	37	4	0
0	0	1	0	1	0	0	0	NA	0	26.69000053	Maxillary	88	43	47	5	0
0	0	1	0	0	0	0	0	NA	0	27.54999924	Maxillary	99	61	40	2	0
1	0	1	0	0	0	0	0	NA	0	27.5	Maxillary	81	59	0	6	0
1	0	1	0	0	0	0	0	NA	0	28.47999954	Ethmoid	81	59	1	2	1
0	0	0	1	0	0	1	1	7	0	17.54000092	Ethmoid	30	8	31	7	1
0	0	0	1	0	1	1	1	6	0	16.57999992	Maxillary	27	6	34	8	1
0	0	0	1	0	0	0	1	23	1	16.57999992	Maxillary	59	64	NA	8	1
1	0	0	1	0	0	0	0	NA	1	30.06999969	Ethmoid	NA	NA	7	NA	NA
0	0	1	0	0	0	0	NA	NA	0	22.01000023	Maxillary	71	19	50	10	0
0	0	1	0	0	1	1	NA	NA	0	22.65999985	Ethmoid	74	23	38	8	1
0	0	1	0	0	1	0	NA	NA	0	24.21999931	Maxillary	68	16	25	6	1
0	0	1	0	0	NA	0	NA	NA	0	24.70999908	Ethmoid	84	32	28	9	1
1	1	1	1	1	0	0	NA	NA	0	19.97999954	Maxillary	76	45	47	8	0
1	1	1	1	1	1	0	0	NA	0	20.02000046	Ethmoid	78	43	47	8	0
1	1	1	1	0	0	0	0	NA	0	19.30999947	Maxillary	73	45	71	9	1
1	1	1	1	0	0	0	0	NA	0	20.12000084	Ethmoid	74	39	53	6	1
1	0	1	0	0	1	0	NA	NA	0	19.59000015	Frontal	62	25	13	8	0
1	0	1	0	0	0	0	1	1	0	18.97999954	Maxillary	59	20	8	6	0
1	0	1	0	0	0	0	0	NA	0	20.04999924	Maxillary	54	17	13	9	1
0	0	0	0	1	1	0	NA	NA	0	34	Ethmoid	65	40	67	10	0
1	0	1	0	1	1	0	NA	NA	0	17.88999939	Ethmoid	51	16	50	12	0
1	0	1	0	0	1	0	1	8	0	17.53000069	Maxillary	54	18	29	8	0
1	0	0	0	0	0	0	0	NA	0	26.25	Maxillary	NA	NA	17	NA	NA
0	0	1	0	0	0	1	0	NA	0	22.11000061	Frontal	34	12	22	9	1

0	0	1	0	0	0	1	0	NA	0	23.04000092	Maxillary	34	8	22	10	0
0	0	1	0	0	0	1	0	NA	0	22.11000061	Ethmoid	32	10	24	10	0
0	0	0	0	0	0	0	0	NA	0	NA	Maxillary	64	36	30	8	1
1	1	1	0	0	0	0	0	NA	0	23.03000069	Maxillary	56	30	58	8	1
1	1	1	0	0	0	0	0	NA	0	24.05999947	Ethmoid	62	31	41	4	1
1	1	1	0	0	0	0	0	NA	0	23.79999924	Ethmoid	56	27	47	4	NA
1	1	1	0	0	0	0	0	NA	0	23.79999924	Maxillary	NA	NA	36	6	NA
1	1	1	0	0	0	0	0	NA	0	23.79999924	Ethmoid	NA	NA	34	6	NA
1	1	1	0	0	0	0	0	NA	0	23.79999924	Ethmoid	NA	NA	34	6	NA
0	0	1	0	0	0	0	0	NA	0	30.47999954	Maxillary	81	63	8	8	1
0	1	0	1	0	0	0	NA	NA	0	22.20000076	Ethmoid	76	11	60	10	1

sputum_staph	sinus_staph	sinus_pa	current_topabx	current_top_vanco	current_top_gent	current_top_mupirocin	current_top_ciprodex	is_subject_on_systemic_abx	IV_vanco	IV_gent	IV_pip-tazo	IV_cephalosporin	IV_carbepenem	IV_colistin	IV_cipro	IV_aztreonam	oral_linezolid	oral_cipro	oral_clinda
NA	0	0	1	1	0	1	0	1	0	0	0	0	0	0	0	0	0	1	0
1	0	0	NA	0	0	0	0	NA	0	0	0	0	0	0	0	0	0	0	0
1	0	0	NA	0	0	0	0	NA	0	0	0	0	0	0	0	0	0	0	0
1	0	0	NA	0	0	0	0	NA	0	0	0	0	0	0	0	0	0	0	0
NA	1	0	1	0	0	1	1	1	0	0	0	0	0	0	0	0	0	1	0
1	1	0	1	1	1	0	0	0	0	0	0	0	0	0	0	0	0	0	0
1	1	0	1	0	1	0	0	1	1	0	0	0	1	0	0	1	0	0	0
1	1	0	1	0	1	1	0	1	1	0	0	0	1	0	0	0	0	0	0
1	1	0	1	0	1	1	0	1	1	0	0	0	0	0	0	0	0	0	0
1	1	0	1	0	1	1	0	1	1	0	0	0	0	0	0	0	0	0	0
1	1	0	1	0	1	1	0	1	1	0	1	0	0	0	0	0	0	0	0
1	1	0	1	0	1	0	0	1	1	0	1	0	0	0	0	0	0	0	0
1	1	0	1	0	1	0	0	1	1	0	1	0	0	0	0	0	0	0	0
1	1	0	0	0	0	0	0	0	0	0	0	0	0	0	0	0	0	0	0
1	1	0	0	0	0	0	0	0	0	0	0	0	0	0	0	0	0	0	0
1	1	0	0	0	0	0	0	0	0	0	0	0	0	0	0	0	0	0	0
1	1	0	0	0	0	0	0	0	0	0	0	0	0	0	0	0	0	0	0
NA	1	0	1	0	0	1	1	1	0	0	0	0	0	0	0	0	0	1	0
0	1	1	1	0	0	1	1	1	0	0	0	0	0	0	0	0	0	1	0
1	1	1	1	0	0	0	1	0	0	0	0	0	0	0	0	0	0	0	0
0	0	0	0	0	0	0	0	0	0	0	0	0	0	0	0	0	0	0	0
NA	1	1	0	0	0	0	0	1	0	0	0	0	0	0	0	0	0	1	0
0	1	1	0	0	0	0	0	0	0	0	0	0	0	0	0	0	0	0	0
1	1	1	0	0	0	0	0	1	0	0	0	0	0	0	0	0	1	0	0
1	1	1	1	0	1	1	0	1	0	0	0	0	0	0	0	0	1	0	0
0	1	1	1	1	1	1	0	1	1	1	0	0	0	0	0	1	0	0	0
1	0	1	1	1	1	0	0	1	1	1	0	0	0	0	0	1	0	0	0
1	1	1	1	1	1	0	0	1	1	1	0	0	0	0	0	1	0	0	1
0	1	1	1	1	1	1	0	1	1	1	0	0	0	0	0	1	0	0	0
1	1	1	1	0	1	1	0	1	1	0	0	0	0	1	0	1	0	0	0
1	1	1	1	0	1	1	0	1	1	0	0	0	0	1	0	0	0	0	0
1	1	1	1	1	1	1	0	1	0	0	0	0	0	1	0	1	0	0	0
1	1	0	1	1	1	1	0	1	1	0	0	0	0	1	0	1	0	0	0
1	1	0	1	0	1	1	0	1	1	0	0	0	0	1	0	1	0	0	0
1	1	0	1	0	1	0	0	1	1	0	0	1	0	1	0	0	0	0	0
NA	0	0	1	0	0	0	0	1	0	0	1	0	0	1	0	0	0	0	0
0	0	0	0	0	0	0	0	1	0	0	0	0	0	0	0	0	0	1	0
0	0	0	0	0	0	0	0	1	0	0	1	0	0	0	0	0	0	0	0
1	1	0	1	0	0	0	0	1	0	0	0	1	0	1	0	0	0	0	0
1	1	0	0	0	0	0	0	1	0	0	1	0	1	0	0	0	0	0	0
0	0	0	0	0	0	0	0	1	0	0	1	0	1	0	0	0	1	0	0
0	1	0	0	0	0	0	0	1	0	0	1	0	1	0	0	0	0	0	0
1	1	0	1	0	0	1	0	1	0	0	0	0	0	0	0	0	0	0	0
1	1	0	0	0	0	0	0	1	1	0	1	0	1	0	0	0	0	0	0
1	1	0	0	0	0	0	0	1	0	0	1	0	1	0	0	0	0	0	0
NA	0	1	0	0	0	0	0	1	0	0	1	1	1	0	0	0	0	1	0
1	0	1	0	0	0	0	0	1	0	0	0	0	0	0	0	0	0	1	0
1	1	1	1	0	1	0	0	1	0	0	0	0	0	0	0	0	1	0	0
1	1	1	0	0	0	0	0	1	0	0	0	0	0	0	0	0	1	0	0
0	0	1	1	0	0	1	0	1	0	0	0	0	0	0	0	0	0	1	0
NA	0	1	1	0	0	1	0	1	0	1	0	0	1	0	0	0	0	0	0
0	0	1	1	0	1	0	0	1	1	1	0	0	0	0	0	1	0	0	0
1	1	1	1	0	1	0	0	1	1	1	1	0	0	0	0	0	0	0	0
0	0	1	1	0	0	1	0	0	0	0	0	0	0	0	0	0	0	0	0
1	0	1	0	0	0	0	0	0	0	0	0	0	0	0	0	0	0	0	0
1	1	0	1	0	0	1	1	1	0	0	0	0	0	0	0	0	0	1	0
NA	1	0	1	0	0	1	0	0	0	0	0	0	0	0	0	0	0	0	0
0	1	1	1	0	0	0	1	1	1	1	1	0	0	0	0	0	0	0	0
0	0	1	1	0	0	1	1	NA	0	0	0	0	0	0	0	0	0	0	0
0	0	0	1	0	0	1	0	NA	0	0	0	0	0	0	0	0	0	0	0
0	1	0	0	0	0	0	0	0	0	0	0	0	0	0	0	0	0	0	0
0	0	0	0	0	0	0	0	0	0	0	0	0	0	0	0	0	0	0	0
1	0	0	0	0	0	0	0	0	0	0	0	0	0	0	0	0	0	0	0
NA	0	0	0	0	0	0	0	0	0	0	0	0	0	0	0	0	0	0	0
1	0	0	0	0	0	0	0	1	0	0	0	0	0	0	0	0	0	0	0
1	0	0	0	0	0	0	0	0	0	0	0	0	0	0	0	0	0	0	0
1	1	0	0	0	0	0	0	1	0	0	0	0	0	0	0	0	0	0	0
0	0	0	1	0	1	0	0	0	0	0	0	0	0	0	0	0	0	0	0
0	0	0	1	0	0	1	0	1	0	0	0	0	0	0	0	0	1	0	0
1	0	0	0	0	0	0	0	0	0	0	0	0	0	0	0	0	0	0	0
0	0	1	0	0	0	0	0	1	0	1	0	0	1	0	0	0	0	0	0
0	0	1	0	0	0	0	0	1	0	0	0	0	1	0	0	0	0	0	0
0	0	1	0	0	0	0	0	1	0	0	0	1	0	1	0	1	0	0	0
NA	0	0	0	0	0	0	0	0	0	0	0	0	0	0	0	0	0	0	0
0	0	0	0	0	0	0	0	1	0	0	0	0	0	0	0	0	0	0	0
1	0	0	0	0	0	0	0	1	0	0	0	0	0	0	0	0	0	0	0
0	1	1	0	0	0	0	0	1	0	0	0	1	1	0	0	0	0	0	0
0	1	1	0	0	0	0	0	1	0	0	0	0	0	0	0	0	0	0	0
1	0	1	0	0	0	0	0	0	0	0	0	0	0	0	0	0	0	0	0
0	0	1	0	0	0	0	0	1	1	1	0	1	0	0	0	0	0	0	0
1	0	1	1	0	0	0	1	0	0	0	0	0	0	0	0	0	0	0	0
1	0	1	1	0	0	0	1	1	0	0	0	0	0	0	0	0	0	1	0
1	1	1	0	0	0	0	0	1	0	0	0	0	0	0	0	0	1	1	0
1	1	0	0	0	0	0	0	1	0	0	0	0	0	0	0	0	1	1	0
1	1	1	0	0	0	0	0	0	0	0	0	0	0	0	0	0	0	0	0
1	1	0	0	0	0	0	0	1	0	0	0	1	0	0	1	0	0	0	0
1	1	0	1	0	0	1	0	0	0	0	0	0	0	0	0	0	0	0	0
0	1	0	1	0	0	1	0	1	1	0	1	0	0	0	0	0	0	0	0
NA	1	0	0	0	0	0	0	0	0	0	0	0	0	0	0	0	0	0	0
0	0	1	0	0	0	0	0	1	0	0	0	0	0	0	0	0	1	1	0

1	1	1	0	0	0	0	0	1	0	0	0	0	0	0	0	0	1	1	0
1	1	1	0	0	0	0	0	1	0	0	0	0	0	0	0	0	1	0	0
0	0	1	0	0	0	0	0	0	0	0	0	0	0	0	0	0	0	0	0
0	0	0	0	0	0	0	0	0	0	0	0	0	0	0	0	0	0	0	0
0	0	1	1	0	0	1	1	1	0	0	0	0	0	0	0	0	0	1	0
NA	1	0	1	0	0	0	1	1	0	1	0	0	0	0	0	0	0	1	0
NA	1	0	1	0	1	0	1	1	0	0	0	0	0	0	0	0	0	0	0
NA	1	1	1	0	1	1	1	1	0	0	0	0	0	0	0	0	0	0	0
NA	1	1	1	0	1	1	0	1	0	0	0	0	0	0	0	0	0	1	0
1	0	1	1	0	1	0	0	0	0	0	0	0	0	0	0	0	0	0	0
0	0	0	0	0	0	0	0	0	0	0	0	0	0	0	0	0	0	0	0

oral_sulfa	oral_doxy	oral_other	oral_blactam	days_IVs_current_course	days_oral_abx_currentcourse	is_patient_currently_on_abx	days_on_current_abx	days_iv_abx_since_last_visit	days_of_oral_since_last_visit	il_1b	il_6	infbeta	il_19	ifn_lambda2	il_29	pentraxin_3
1	1	0	0	NA	112	1	112	0	112	1060.17	6.9	15.24	7.85	4.93	12.74	1192.07
0	0	0	0	NA	NA	NA	NA	NA	NA	NA	NA	NA	NA	NA	NA	NA
0	0	0	0	NA	NA	NA	NA	NA	NA	NA	NA	NA	NA	NA	NA	NA
0	0	0	0	NA	NA	NA	NA	NA	NA	NA	NA	NA	NA	NA	NA	NA
0	1	0	1	NA	NA	0	NA	0	28	NA	NA	NA	NA	NA	NA	NA
0	0	0	0	NA	NA	0	NA	NA	NA	NA	NA	NA	NA	NA	NA	NA
0	0	0	0	17	NA	1	17	49	0	1069.06	6.9	14.95	11.24	7.24	18.37	960.69
0	0	0	0	23	NA	1	23	35	0	49.38	2.14	14.37	14.24	2.6	12.74	637.03
0	0	0	0	NA	NA	0	NA	7	0	111.5	15.4	6.18	282.31	8.01	1.17	2351.7
1	0	0	0	0	0	0	NA	30	28	3164.37	9.31	16.69	9.36	6.47	15.56	1742.14
0	0	0	0	0	NA	0	NA	25	0	NA	NA	NA	NA	NA	NA	NA
0	0	0	0	14	NA	1	14	32	0	NA	NA	NA	NA	NA	NA	NA
1	0	0	0	0	0	0	NA	7	14	NA	NA	NA	NA	NA	NA	NA
0	0	0	0	NA	NA	0	NA	42	NA	114.62	40.62	10.87	213.93	4.54	14.16	2526.53
0	0	0	0	NA	NA	0	NA	NA	NA	NA	NA	NA	NA	NA	NA	NA
0	0	0	0	NA	NA	0	NA	0	0	615.05	80.24	14.95	16.61	5.7	21.17	1821.09
0	0	0	0	NA	NA	0	NA	0	0	1862.1	47.75	11.46	452.13	6.47	19.77	2009.02
0	0	0	0	NA	8	1	8	0	8	NA	NA	NA	NA	NA	NA	NA
0	0	0	0	NA	NA	0	NA	0	14	NA	NA	NA	NA	NA	NA	NA
0	0	0	0	NA	NA	0	NA	0	0	NA	NA	NA	NA	NA	NA	NA
0	0	0	0	NA	NA	0	NA	NA	NA	NA	NA	NA	NA	NA	NA	NA
0	1	0	0	NA	NA	0	NA	14	14	2128.04	9.31	16.98	15.05	7.24	21.17	1358.57
0	0	0	0	NA	NA	0	NA	24	84	NA	NA	NA	NA	NA	NA	NA
0	1	0	0	NA	NA	0	NA	0	35	7.38	9.72	14.95	8.87	4.15	9.9	95.39
1	1	0	0	NA	56	1	56	0	56	NA	NA	NA	NA	NA	NA	NA
0	0	0	0	NA	NA	0	NA	21	0	6137.29	33.94	17.84	23.69	8.01	12.74	1695.74
0	0	0	0	NA	NA	0	NA	0	0	48	7.7	12.04	10.78	3.38	9.9	699.34
0	1	1	0	NA	NA	0	NA	20	14	963.52	8.91	17.27	27.22	6.86	15.56	1400.09
0	0	0	0	12	NA	1	12	12	0	106.38	26.48	6.77	1569.81	4.15	4.14	2165.42
0	0	0	0	NA	NA	0	NA	44	6	2813.23	17.23	9.71	18.85	4.93	4.14	1567.41
0	0	0	0	22	NA	1	22	22	14	NA	NA	NA	NA	NA	NA	NA
0	0	0	0	NA	NA	0	NA	44	0	NA	NA	NA	NA	NA	NA	NA
0	0	0	0	NA	NA	0	NA	0	0	NA	NA	NA	NA	NA	NA	NA
0	0	0	0	20	NA	1	20	20	0	NA	NA	NA	NA	NA	NA	NA
0	0	0	0	NA	NA	0	NA	35	0	NA	NA	NA	NA	NA	NA	NA
0	0	0	0	16	NA	1	16	65	0	278.7	14.99	19.58	14.24	6.47	18.37	945.22
0	1	0	0	NA	NA	0	NA	0	28	801.7	59.57	19	27.53	7.63	21.17	1726.63
0	1	0	0	NA	NA	0	NA	18	18	154.64	12.55	23.33	20.98	8.78	23.95	1558.75
0	0	0	0	16	NA	NA	NA	NA	NA	910.53	21.12	19	75.4	7.24	23.95	2269.72
0	1	0	0	NA	NA	0	NA	48	48	839.66	9.52	17.27	9.85	5.7	18.37	1046.28
0	0	0	0	9	9	1	9	9	41	NA	NA	NA	NA	NA	NA	NA
0	0	0	0	NA	NA	0	NA	10	10	NA	NA	NA	NA	NA	NA	NA
0	1	0	0	NA	NA	0	NA	0	0	NA	NA	NA	NA	NA	NA	NA
0	0	0	0	10	NA	1	10	10	10	NA	NA	NA	NA	NA	NA	NA
0	0	0	0	NA	NA	0	NA	15	15	NA	NA	NA	NA	NA	NA	NA
0	0	0	0	NA	NA	0	NA	35	0	NA	NA	NA	NA	NA	NA	NA
0	0	0	0	NA	NA	0	NA	0	0	4089.93	751.02	62.92	2981.32	554.38	33.63	4170.55
1	0	0	0	NA	6	1	6	0	20	797.35	12.55	3.22	1212.73	3.38	12.74	1548.23
1	0	0	0	NA	NA	0	NA	0	10	143.77	11.13	4.7	180.71	4.15	9.9	991.1
0	0	0	0	NA	NA	0	NA	0	14	110.02	11.74	5	69.47	4.15	11.32	456.37
0	0	0	0	NA	NA	0	NA	8	14	1341.94	6.1	7.36	11.24	5.7	12.74	1357.12
0	0	0	0	NA	NA	0	NA	10	14	NA	NA	NA	NA	NA	NA	NA
0	0	0	0	NA	NA	0	NA	0	0	NA	NA	NA	NA	NA	NA	NA
0	0	0	0	NA	NA	0	NA	0	NA	6.66	1.36	11.46	39.05	1.81	7.04	158.85
0	0	0	0	NA	NA	0	NA	NA	NA	486.93	23.59	2.62	3321.42	4.15	4.14	771.85
0	1	0	0	NA	0	1	0	0	NA	1310.68	451.58	11.75	20802.16	13.37	32.26	1897.94
0	0	0	0	NA	NA	0	NA	14	NA	3.34	2.93	12.04	187.18	2.6	7.04	119.27
0	0	0	0	NA	NA	1	21	21	NA	19.27	14.99	18.42	12.56	5.7	15.56	430.91
0	0	0	0	NA	NA	0	NA	28	NA	84.66	20.3	5.59	1718.51	3.38	7.04	741.11
0	0	0	0	NA	NA	0	NA	0	NA	NA	NA	NA	NA	NA	NA	NA
0	0	0	0	NA	NA	0	NA	0	NA	NA	NA	NA	NA	NA	NA	NA
0	0	0	0	NA	NA	0	NA	NA	NA	NA	NA	NA	NA	NA	NA	NA
0	0	0	0	NA	NA	0	NA	0	0	823.65	8.51	14.37	8.87	6.09	15.56	953.96
0	0	0	0	NA	NA	0	NA	0	0	46.34	5.9	17.27	9.85	4.93	15.56	506.79
1	0	0	0	NA	NA	0	NA	0	14	34.98	19.48	12.04	733.37	5.7	21.17	2173.76
0	0	0	0	NA	NA	0	NA	0	28	97.56	25.65	6.18	203.53	3.38	9.9	1107.58
1	0	0	0	NA	NA	0	NA	0	14	NA	NA	NA	NA	NA	NA	NA
0	0	0	0	NA	NA	0	NA	0	0	NA	NA	NA	NA	NA	NA	NA
1	1	0	0	NA	0	0	NA	0	42	757.86	9.11	14.08	7.85	4.15	11.32	1349.87
0	0	0	0	NA	NA	0	NA	0	NA	219.42	12.95	5.59	177.6	4.15	1.17	1213.23
0	0	0	0	NA	NA	0	NA	50	NA	70.63	13.16	3.22	578.83	3.38	980.28	1140.24
0	0	0	0	6	NA	1	6	6	14	NA	NA	NA	NA	NA	NA	NA
0	0	0	0	NA	NA	0	NA	54	0	NA	NA	NA	NA	NA	NA	NA
0	0	0	0	NA	NA	0	NA	NA	NA	NA	NA	NA	NA	NA	NA	NA
0	0	0	1	NA	NA	0	NA	NA	NA	12024.87	8.91	13.2	10.78	6.47	18.37	1360.02
0	0	0	1	NA	19	1	19	0	40	1413.6	8.91	12.62	18.85	7.24	15.56	1891.56
0	0	0	1	NA	NA	0	NA	26	14	7076.08	21.33	10.29	15.84	8.01	15.56	1930.72
0	1	0	1	NA	NA	0	NA	0	42	NA	NA	NA	NA	NA	NA	NA
0	0	0	0	NA	NA	0	NA	NA	NA	24.99	4.51	7.36	346.04	3.38	9.9	543.2
0	0	0	0	40	NA	1	40	40	28	6.35	8.11	0.19	1681.65	4.15	9.9	292.89
0	0	0	0	NA	NA	0	NA	7	0	0.06	1.36	12.04	7.85	1.02	7.04	53.7
0	1	1	0	NA	NA	0	NA	0	28	NA	NA	NA	NA	NA	NA	NA
0	0	0	0	NA	20	1	20	NA	NA	32.95	15.8	24.76	20.28	14.13	43.23	861.27
0	1	0	0	NA	NA	0	NA	0	45	366.58	8.71	14.37	7.85	4.93	12.74	1267.9
0	0	0	0	NA	NA	0	NA	21	NA	NA	NA	NA	NA	NA	NA	NA
0	0	0	0	NA	NA	1	6	NA	NA	1006.48	20.3	13.2	18.85	7.24	9.9	1438.6
0	0	0	0	NA	NA	0	NA	NA	NA	334.56	8.11	21.02	275.13	5.7	12.74	1051.07
0	0	0	0	NA	NA	0	NA	16	14	66.85	36.44	12.62	10.78	4.93	9.9	1272.19
0	0	0	0	NA	NA	0	NA	NA	NA	NA	NA	NA	NA	NA	NA	NA
0	1	0	0	NA	NA	NA	NA	NA	NA	94.12	12.14	9.12	13.41	5.7	15.56	751.44

0	1	0	0	NA	NA	NA	NA	NA	NA	NA	NA	NA	NA	NA	NA	NA
1	1	0	0	NA	NA	NA	NA	NA	NA	NA	NA	NA	NA	NA	NA	NA
0	0	0	0	NA	NA	0	NA	NA	NA	1824.57	10.12	16.69	26.28	7.24	18.37	1729.73
0	0	0	0	NA	NA	0	NA	NA	NA	342.72	12.14	19.58	15.05	8.78	32.26	1085.46
0	0	0	0	NA	NA	0	NA	0	28	1528.44	12.14	17.84	15.84	8.01	18.37	1128.4
0	0	0	0	0	0	0	NA	13	14	NA	NA	NA	NA	NA	NA	NA
0	1	0	0	NA	NA	0	NA	0	14	NA	NA	NA	NA	NA	NA	NA
1	0	0	0	NA	NA	0	NA	0	14	NA	NA	NA	NA	NA	NA	NA
0	0	0	0	NA	NA	1	10	0	24	NA	NA	NA	NA	NA	NA	NA
0	0	0	0	NA	NA	0	NA	NA	NA	368.23	10.73	13.79	23.02	3.38	9.9	772.83
0	0	0	0	NA	NA	0	NA	NA	NA	NA	NA	NA	NA	NA	NA	NA

Supplemental table 5. Codebook describing each variable in the metadata. See Supplemental table 4 for the metadata.

Variable	Description
SampleID	Unique name for the 16S amplicon sequencing sample
BarcodeSequence	Sequence of the barcode
LinkerPrimerSequence	Sequence of the linker primer
participant_id	The study participant's unique ID number
visit_number	Numerical order of visits for each participant (ascending)
days_since_first_visit	Count of the number of visits since enrollment (Day 0)
sex_is_female	male = 0, female = 1
age_onenrollment	in years
BMI_on_enrollment	mg/kg2
cfrd	prior diagnosis of CF-related diabetes; 0 = no, 1 = yes
transplant_prior_to_enrollment	prior LUNG transplant at day of enrollment, no = 0, yes = 1. Note that one subject gets a transplant during the study
hmzg_mut508	homozygous = 0, heterozygous = 1, other mutations = 2, missing = NA
allergic_rhinitis	no = 0, yes = 1
ever_on_topabx	Is the subject on topical antibiotics at ANY TIME during the study, no = 0, yes = 1
ever_on_topvanco	If the subject on specific topical antibiotics at ANY TIME during the study, no = 0, yes = 1
ever_on_topgent	If the subject on specific topical antibiotics at ANY TIME during the study, no = 0, yes = 1
ever_on_top_mupirocin	If the subject on specific topical antibiotics at ANY TIME during the study, no = 0, yes = 1
ever_on_top_cipro	If the subject on specific topical antibiotics at ANY TIME during the study, no = 0, yes = 1
ever_on_nasal_steroid	Is the subject on a nasal steroid at ANY TIME during the study? no = 0, yes = 1
ever_on_chronic_oral_steroid	Is the subject on chronic oral prednisone during the study? no = 0, yes = 1
sinus_exacerbation	Is this an unscheduled visit because of worse sinus disease? no = 0, yes = 1
pulmonary_exacerbation	Have they been treated for a CF pulmonary exacerbation in the month surrounding the study visit (+/- 4 weeks on each side)? no = 0, yes = 1
on_nasal_cannula_oxygen	no = 0, yes = 1
interim_hos	no = 0, yes = 1
hospital_days	if yes to interim_hospitalization, then this is the number of days in the hospital since the last visit
transplant_at_visit	transplant status on DAY OF VISIT no = 0, yes = 1
visit_bmi	mg/kg2
samp_loc	the site in the sinus cavity where the sample taken from
ppFEV1	% predicted FEV1 (theoretical max is about 110%, min around 25%)
ppFEF25-75	% predicted FEF25-75
snot22	sinus symptom scale 0-100. people without sinus disease score 0-7 points
mlk	endoscopy visual severity score, modified lund kennedy scale, normal = 0, maximally severe disease = 16
sputum_pa	Was Pseudomonas aeruginosa grown from the sputum at this visit (or within 1 month, from the medical records); n = 0, yes = 1.
sputum_staph	Was Staphylococcus aureus grown from the sputum at this visit (or within 1 month, from the medical records); n = 0, yes = 1.
sinus_staph	Was Pseudomonas aeruginosa grown from the sinuses at this visit (or within 1 month, from the medical records); n = 0, yes = 1.
sinus_pa	Was Staphylococcus aureus grown from the sinuses at this visit (or within 1 month, from the medical records); n = 0, yes = 1.
current_topabx	is the subject currently on topical sinus rinses, no = 0, yes = 1
current_top_vanco	if the subject is on rinses, which drug, no = 0, yes = 1
current_top_gent	if the subject is on rinses, which drug, no = 0, yes = 1
current_top_mupirocin	if the subject is on rinses, which drug, no = 0, yes = 1
current_top_ciprodex	if the subject is on rinses, which drug, no = 0, yes = 1
is_subject_on_systemic_abx	is the subject currently on systemic antibiotics (oral or IV). From retrospective chart review.
IV_vanco	if the subject is on systemic antibiotics, which one, no=0, yes=1, these are by drug class. From retrospective chart review.
IV_gent	if the subject is on systemic antibiotics, which one, no=0, yes=1, these are by drug class. From retrospective chart review.
IV pip-tazo	if the subject is on systemic antibiotics, which one, no=0, yes=1, these are by drug class. From retrospective chart review.
IV_cephalosporin	if the subject is on systemic antibiotics, which one, no=0, yes=1, these are by drug class. From retrospective chart review.
IV_carbepenem	if the subject is on systemic antibiotics, which one, no=0, yes=1, these are by drug class. From retrospective chart review.
IV_colistin	if the subject is on systemic antibiotics, which one, no=0, yes=1, these are by drug class. From retrospective chart review.
IV_cipro	if the subject is on systemic antibiotics, which one, no=0, yes=1, these are by drug class. From retrospective chart review.
IV_aztreonam	if the subject is on systemic antibiotics, which one, no=0, yes=1, these are by drug class. From retrospective chart review.
oral_linezolid	if the subject is on systemic antibiotics, which one, no=0, yes=1, these are by drug class. From retrospective chart review.
oral_cipro	if the subject is on systemic antibiotics, which one, no=0, yes=1, these are by drug class. From retrospective chart review.
oral_clinda	if the subject is on systemic antibiotics, which one, no=0, yes=1, these are by drug class. From retrospective chart review.
oral_sulfa	if the subject is on systemic antibiotics, which one, no=0, yes=1, these are by drug class. From retrospective chart review.
oral_doxy	if the subject is on systemic antibiotics, which one, no=0, yes=1, these are by drug class. From retrospective chart review.
oral_other	if the subject is on systemic antibiotics, which one, no=0, yes=1, these are by drug class. From retrospective chart review.
oral_blactam	if the subject is on systemic antibiotics, which one, no=0, yes=1, these are by drug class. From retrospective chart review.
days_IVs_current_course	days of IV therapy as of visit in current course. From retrospective chart review.
days_oral_abx_currentcourse	days of oral therapy as of visit in current course. From retrospective chart review.
is_patient_currently_on_abx	is the subject on IV abx AT THE DAY OF VISIT
days_on_current_abx	days on antibitiotics as of the visit
days_iv_abx_since_last_visit	total number of days on IV abx since last visit
days_of_oral_since_last_visit	total number of days on oral abx since last visit.
il_1b	continuous variable (pg/mL)
il_6	continuous variable (pg/mL)
infbeta	continuous variable (pg/mL)
il_19	continuous variable (pg/mL)
ifn_lambda2	continuous variable (pg/mL)
il_29	continuous variable (pg/mL)
pentraxin_3	continuous variable (pg/mL)
